# A threshold-free model of numerosity comparisons

**DOI:** 10.1371/journal.pone.0195188

**Published:** 2018-04-05

**Authors:** Santiago Alonso-Diaz, Jessica F. Cantlon, Steven T. Piantadosi

**Affiliations:** Department of Brain and Cognitive Sciences, University of Rochester, Rochester, NY, United States of America; Uppsala Universitet, SWEDEN

## Abstract

A dominant mechanism in the Judgment and Decision Making literature states that information is accumulated about each choice option until a decision threshold is met. Only after that threshold does a subject start to execute a motor response to indicate their choice. However, recent research has revealed spatial gradients in motor responses as a function of comparison difficulty as well as changes-of-mind in the middle of an action, both suggesting continued accumulation and processing of decision-related signals after the decision boundary. Here we present a formal model and supporting data from a number comparison task that a continuous motor planner, combined with a simple statistical inference scheme, can model detailed behavioral effects without assuming a threshold. This threshold-free model reproduces subjects’ sensitivity to numerical distance in reaching, accuracy, reaction time, and changes of mind. We argue that the motor system positions the effectors using an optimal biomechanical feedback controller, and continuous statistical inference on outputs from cognitive processes.

## Introduction

Numbers are core cognitive representations, detectable across species and early in development [1, 2]. The abstract nature of number makes it ideal as an information source and a decision tool. A baboon for example can check the heading direction of its troop by checking how many members move in a given direction [3]. The mechanism to compare numerical values has mainly been modeled with the idea that agents accumulate evidence and commit to a course of action only once a decision threshold has been met [4, 5]. Threshold models (single or multiple stage) can account for detailed behavioral patterns across tasks including reaction times and accuracies observed empirically [4–10], and have been successfully applied in the domain of number cognition [2, 11, 12]. However, they do not easily capture all phenomena, including graded motor attraction to non-targets during movement [13, 14], which suggest that information processing continues after a decision is made. Moreover, changes of mind, defined as transient commitments to one option before suddenly turning to another, are not predicted because, in principle, a threshold was hit and the theory requires ad hoc additions to explain the presence of movement deflections [15]. Threshold models have also been criticized for being unable to capture subjects’ fast appraisal of their environment and task demands [16]. In fact, it seems too slow of an strategy to wait for thresholds in natural environments with non-stationary properties (e.g. a river) in which categorical responses (e.g. move left) might not be appropriate. For example, a salmon in a river with strong currents might be better off continuously using information from its surroundings in a dynamical feedback system, than thresholding each of its individual control actions.

**Fig 1:**
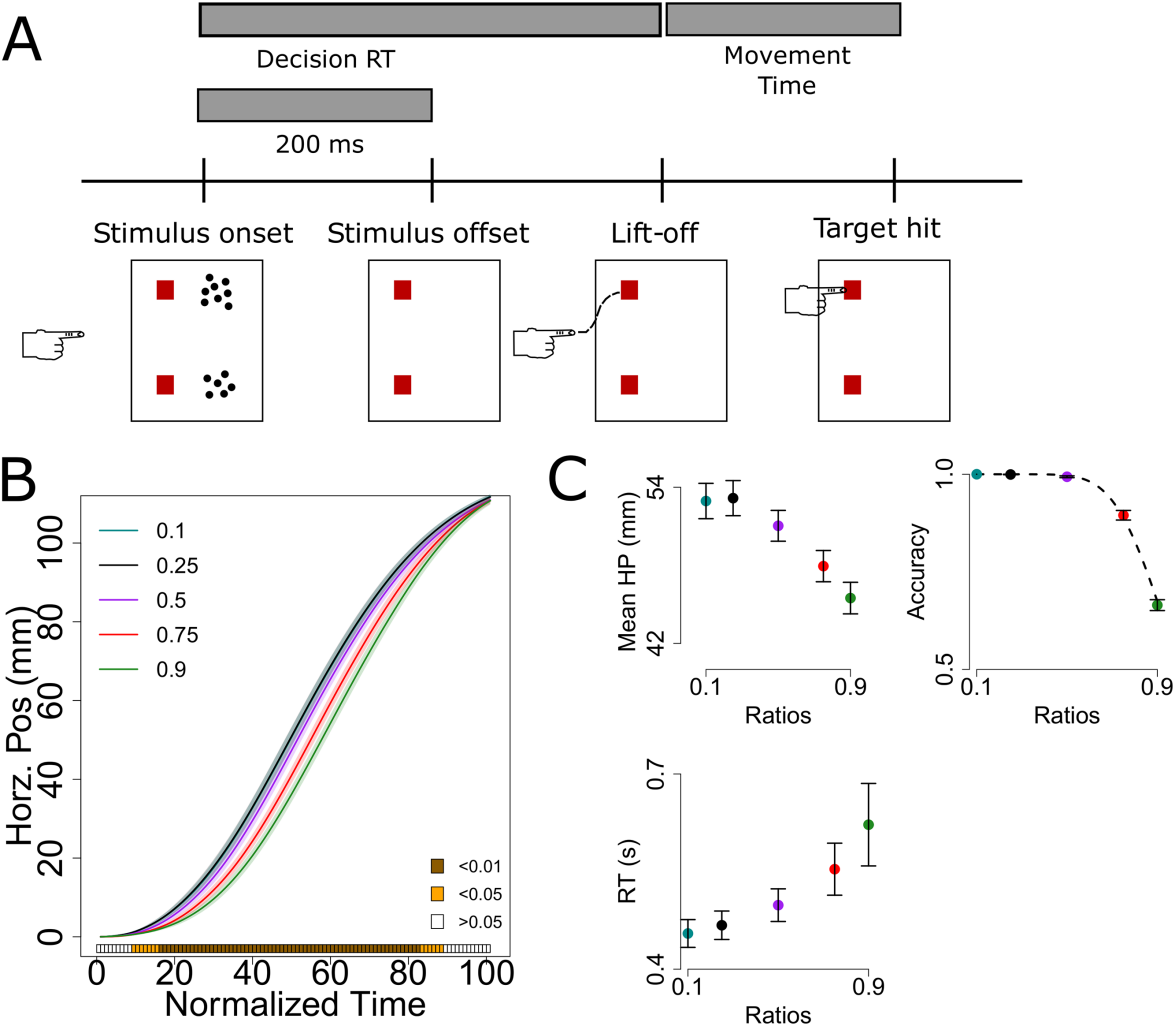
Task (A), reach gradient (B), and discrete behavioral measures (C). (**A**) Subjects were asked to report the more numerous array of dots by moving their index finger from a starting position to one of two targets on screen representing each array. The number of the arrays differed by fixed ratios; for instance a 0.1 ratio could be a trial with 1 dot vs 10 dots. The maximum number of dots on a given array was 25. (**B**) Average subjects’ reach revealed an organized gradient as a function of numerical ratio. The heat bar below represents significance of number ratio effects at each time step, estimated with functional ANOVAs [19]. (**C**) Accuracy and response times were sensitive to numerical ratio. Dashed line in accuracy plot was fitted with Eq 2: Weber fraction = 0.17. Mean horizontal positions along the trajectory (HP) are also shown as a discrete summary of the trajectories. For readers with poor access to color information (e.g. black and white print or colorblindness), all lines and points in this and the remaining figures are mostly organized according to numerical ratio: in plots with horz. positions easier ratios were more positive; in accuracy plots easier ratios were more accurate; in RT plots easier ratios were faster (also the x-axis is usually depicting numerical ratio). Shading and error bars are s.e.m.

An alternative paradigm for understanding action is that evidence is used continuously and behavior develops along the output of an inference over the evidence stream. To test this idea, we analyzed data from a number comparison task in which participants were asked to compare two arrays of dots that differed in number. Their task was to point to the more numerous array by displacing the index finger from a distant starting position to one of two targets on a computer screen. During the choice response, subjects’ reach trajectories were spatially modulated by the difficulty of the number comparison. Reaching responses were sensitive to the numerical distance, with closer numbers generating positions proximal to the midline between the two targets (Fig 1). The results indicated that number processing occurs during the motor response, along the reaching trajectory [17].

With the purpose of explaining the empirical results, we developed a *threshold-free* movement model in which an optimal motor planner [18] was combined with continuous statistical inference about the correct target for a movement. Because the model is threshold free, movement effectively “starts” immediately after the first units of evidence start to arrive, but slowly at the beginning due to high uncertainty. As evidence accumulates, certainty increases and the effector heads to the best target. With such a framework we reproduced signature behavioral findings, including the sensitivity of motor positioning, accuracy, and reaction times to numerical distance. In the next sections we present the model and argue that it provides a parsimonious account of numerical decision-making and motor responses modulated by cognitive output. Furthermore, the use of a numerical task is incidental in the sense that this is not an investigation on the nature of mental number representation, say log. vs. linear, but of a decision mechanism. Therefore, the presented framework is potentially applicable to other judgments and decisions and it raises the novel possibility that decision thresholds are unnecessary in other tasks once models of motor planning implement statistical inference in decision-making.

## Experiment

### Materials and methods

#### Ethical statement

Experimental procedures adhered to university and national ethics standards, as approved by the Research Subjects Review Board at the University of Rochester. All participants provided written consent to participate in the study.

#### Participants

22 right-handed participants (13 female; Mean age: 20.5 yrs, SD: 2.2 yrs) participated in the experiment.

#### Task and apparatus

Participants were asked to report the side with more dots by reaching to large clear targets on a screen with their index finger (Fig 1). At each trial, movement started ∼29 cm from the screen, with the index finger pressing down on a start button. Dots stayed on-screen for 200 ms and subjects were free to release the button anytime and reach to the desired target. Flight time was not enforced and no performance feedback was provided.

Five numerical ratios were used (0.1, 0.25, 0.5, 0.75 and 0.9) equally distributed among 420 trials (presented in 4 blocks). For example, a ratio of 0.1 is a trial with 1 dot vs. 10 dots (we used maximum 25 dots). The side with more dots was counterbalanced. In half of the trials both sides had equal cumulative area covered by the dots (this is the area of each dot summed over all dots), intended to discourage the use of this visual cue. Also, by randomly placing the dots a density strategy was thwarted. In general, it is well stablished that people can use visual number over other cues [1, 20–22].

Recording was done with a Northern Digital Optotrak 3020, sampling at 200 Hz, and stopped 4 cm from the screen. This was mostly for technical reasons i.e. avoid signal loss when approaching the screen. Trajectories where subjects pulled back during flight were not analyzed (7.66% dropout) and only correct trials were considered (see reach in incorrect trials in S9 Fig). A correct trial was defined as a trajectory with 50% or more of points, including the end point, on the appropriate response side as determined by the midline of the screen (90% of trajectories). Therefore, any observed reaching modulation is related to motor plans that were heading toward the correct target most of the time. To have the same number of points for all trajectories, time was normalized to an arbitrary number of points (101) [17, 23–25]. Left and right trajectories were averaged by flipping the sign of the left trajectory (see left and right reach separately in S2 Fig). Results focused on the horizontal axis as the response targets were placed laterally, and number ratio effects were visibly dampened in the depth coordinate (S4 Fig).

## Model: Evidence and controller

**Fig 2:**
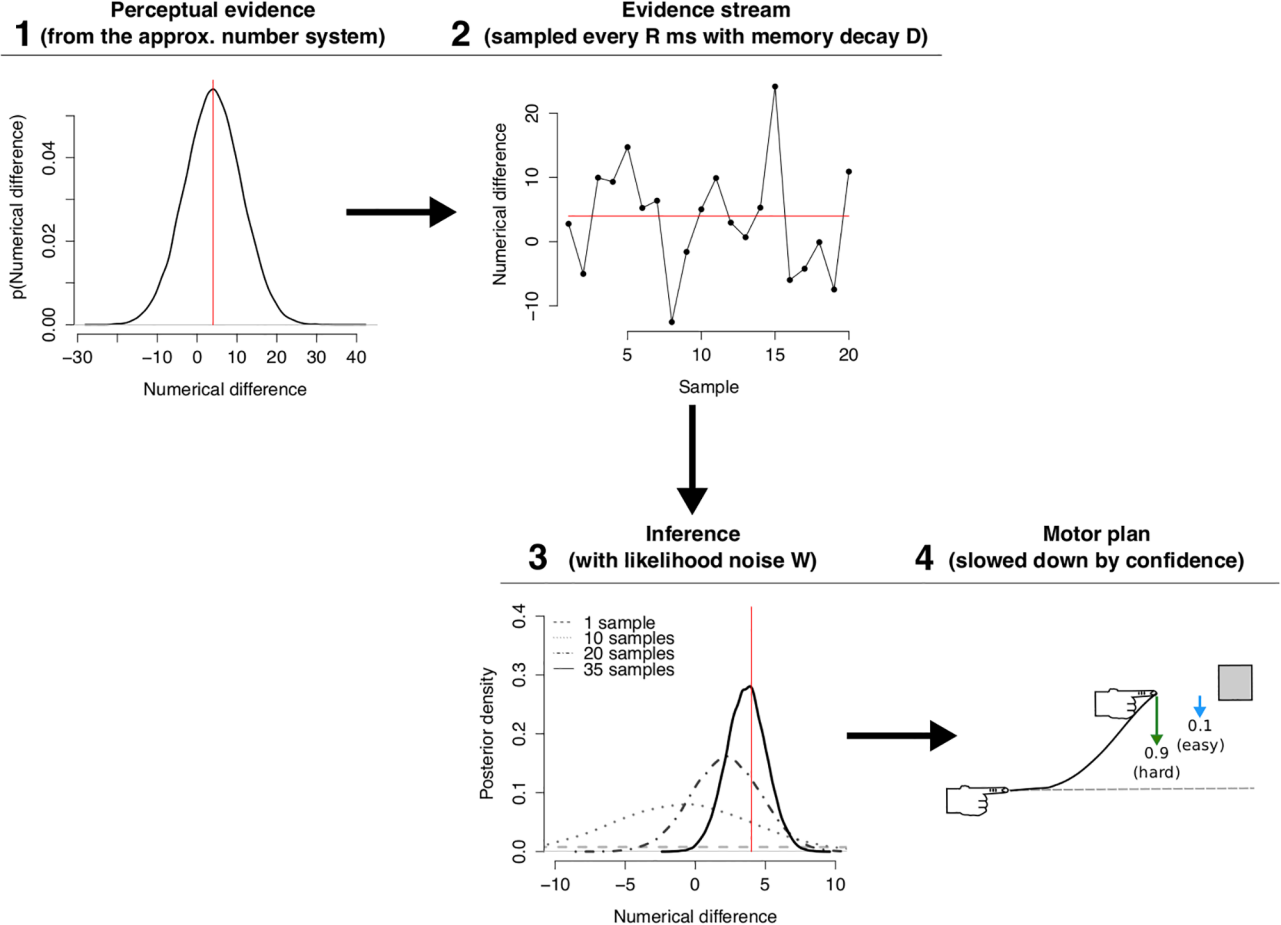
Model diagram. (**1**) The approximate number system produces evidence/samples at a rate R e.g. every 30 ms. (**2**) This generates a stream of evidence. The importance of each sample decays with a rate D (S1 Fig). The decay accounts for memory effects in participants. (**3**) Instead of summing the stream to a threshold, evidence is used to infer which target has the larger number. As more samples arrive the width or confidence on the estimation improves. (**4**) The index finger is placed by a motor controller that minimizes movement jerk and that is slowed down by the degree of confidence. The green and cyan arrows symbolize the hardest and easiest numerical ratio, respectively. Their length depict degree of uncertainty; e.g. harder comparisons produce stronger pulls to the other response target. The red line across panels is the veridical numerical difference.

The model accumulated evidence, executed statistical inference on the potential target of behavior given the evidence, and positioned the motor effector with an optimal feedback controller. This set up allowed us to explore plausible cognitive variables involved in a decision-making task that requires a number judgment: sample rate (*R*), sample memory decay (*D*), and acuity of the Bayesian estimate of the numerical difference (*W*). The general picture we describe is that the participants accumulate evidence about which choice is the correct one, weight this evidence according to a memory decay, and feed their estimates and uncertainty into a continuously-operating motor controller in order to move (Fig 2). Note that the term “accumulation” is not referring to the accumulator model in number cognition literature. We understand accumulation as the process of obtaining and recording samples from an internal representation (see below for details) while the number cognition accumulator refers to a model of non-verbal counting [26].

### Evidence

Noisy evidence was sampled from the internal representation of the numerical difference. The internal representation was assumed Gaussian, following standard psychophysics of number [27, 28],

$$\begin{array}{c} s\left(i\right)\;∼Normal(|n_{\text{L}}-n_{\text{R}}|,\omega \sqrt{n_{\text{L}}^{2}+n_{\text{R}}^{2}}) \end{array}$$ (1)

s(i) is the sample i, taken every *R* ms (a free parameter). The symbol ∼ stands for “distributed as”. *n*_L_ and *n*_R_ are the presented numbers on each side. Samples formed the evidence stream *s* = (*s*_1_, *s*_2_, *s*_3_,…, *s*_*i*_,…, *s*_*n*_), with *n* denoting the total number of samples at a given movement time *t*. The values for *R* were bounded to be between 1-100 ms to reflect realistic values of spiking rates of number processing neurons [29]. A negative sample from Eq (1) is evidence favoring the wrong number.

The value of *ω* determines evidence noise (Eq (1)). Following number cognition literature it will be referred to as the Weber fraction and it is computed by fitting average error rate across participants to,

$$\begin{array}{c} \frac{1}{2}erfc(\frac{|n_{\text{L}}-n_{\text{R}}|}{\omega \sqrt{2}\sqrt{n_{\text{L}}^{2}+n_{\text{R}}^{2}}}) \end{array}$$ (2)

*erfc* is the complementary error function [30]. A Weber fraction was computed for each participant. The obtained mean Weber fraction from participants’ choices was equal to 0.17 (s.d. = 0.04; mean *R*^2^ = 0.96, s.d. = 0.02), in line with previous number comparison experiments [31]. We also computed a Weber fraction using all the data and the obtained value was also around 0.17. In the model we used the latter.

The numerical stimulus was on-screen for a brief time which means that evidence samples were produced from memory (see sampling from memory in [32]). We introduced a memory decay to the evidence stream s in order to model a upweighting of initial information in the statistical inference. We used an exponential moving average (EMA) [33]

$$\begin{array}{c} EMA(i)=D\cdot s(i)+(1-D)\cdot EMA(i-1) \end{array}$$ (3)

We initialize *EMA*(0) = *s*(1). *D* is a free parameter between 0 and 1 that determines the strength of the memory decay. For example, if *D* = 1 the evidence stream s is unmodified (no memory of previous samples). Alternatively, a *D* close to 0 makes the series gravitate around the initial sample, akin to a system with primacy effects in memory [34] (for visualizations of how the memory decay parameter works see S1 Fig). Other way of interpreting *D* is as a generalization of different accumulation schemes. A *D* = 1 is a memory-free stochastic accumulator in which only the noise of the sampling determines choice [35]. A *D* = 0 is a non-stochastic linear ballistic accumulator [10] in that sampling noise is completely reduced by the initial sample. Values in between can be regarded as continuous variations of these possibilities.

### Controller and statistical inference

The model used an optimal feedback control, a framework that has been successful at explaining a wide array of motor behavior [36]. In general, this framework proposes that motor commands try to minimize a cost function along the movement, while concurrently taking into account the present state of the system and task demands. This flexibility was essential for our purposes of generating movements that were affected by continuous number processing.

Specifically, we tackle the kinematic problem with a criterion of jerk (third derivative of position) minimization [37] and an optimal feedback controller derived by [18] (refer to those works for further details on the analytical derivations). At each time step *t*, changes to the state *q* of the motor effector were determined by,

$$\begin{array}{c} \dot{q}\left(t\right)=[\begin{array}{c} \dot{x}\\ \ddot{x}\\ \dddot{x} \end{array}]=[\begin{array}{ccc} 0 & 1 & 0\\ 0 & 0 & 1\\ \frac{-60}{\delta^{3}} & \frac{-36}{\delta^{2}} & \frac{-9}{\delta } \end{array}][\begin{array}{c} x\\ \dot{x}\\ \ddot{x} \end{array}]+[\begin{array}{c} 0\\ 0\\ \frac{60}{\lambda^{3}} \end{array}]x_{f} \end{array}$$ (4)

The controller used the current position *x*, velocity $\dot{x}$, acceleration $\ddot{x}$, the remaining time *δ* = *t*_*f*_ − *t*, and the end target *x*_*f*_, to update the effector state. Here *t* refers to current movement time and *t*_*f*_ to total movement time. Movement time *t*_*f*_ was randomly sampled at the beginning of each simulated trial from the data of the appropriate ratio. Even though fixed movement times are not ideal, spontaneously generating them is a difficult problem and many influential motor models use a similar strategy of deriving them from data [18, 23, 37, 38]. For the interested reader, derivations of optimal movement time for the jerk model can be found in [39]. The kinematic state *q* was initialized at 0.

Recent work suggests that motor plans are modulated by certainty [40], an assumption that we incorporate and extend. The model first determined the probability of which side had the largest number of dots with the cumulative probability,

$$p_{\text{L}}=p(\mu >0),$$ (5)

$$p_{\text{R}}=1-p_{\text{L}}$$ (6)

with *μ* = *n*_L_ − *n*_R_ i.e. the numerical difference. If the left side has the larger number, Eq (5) represents the probability of being correct.

The probabilities *p*_L_ and *p*_R_ evolved according to the sampled numbers. More concretely, we assumed that subjects were trying to position the effector with the best possible estimate of the numerical difference *μ* = *n*_L_ − *n*_R_, given all the samples *e* in *EMA* taken up to time *t*. The numerical difference *μ* was inferred with a uniform prior *p*(*μ*), and normal likelihood *p*(*e*|*μ*, *W*, *n*_1_, *n*_2_,); with *W* as a free parameter representing the likelihood noise. The resulting posterior *p*(*μ*|*W*, *n*_1_, *n*_2_, *e*) from a uniform prior and normal likelihood is itself normally distributed (due to space considerations we direct the reader interested in this derivation to [41]),

$$\begin{array}{c} p(\mu |W,n_{1},n_{2},e)∼N(\bar{e},\frac{W}{n}) \end{array}$$ (7)

where, $\bar{e}$ is the mean of the EMA samples. The free parameter *W* determines the overall confidence in the obtained mean numerical difference. It should not be confused with *ω* above, which determines the noise of the internal numerical samples: *ω* characterizes sampling in perception, while *W* characterizes confidence in action. Conceptually, Eq 7 tells us that the Bayesian estimate of *μ* is centered at the mean of the samples *e* and the noise of that estimate (W) is reduced as more samples (n) arrive. In more direct terms, a Bayesian agent should be more confident of their numerical perception as they get more evidence.

Heading direction was then determined with the largest probability e.g. if *p*_L_ ≥ *p*_R_, move to the left. The certainty was defined to be a rescaling of the entropy over *p*_L_ and *p*_R_ that gives a measure between 0 and 1:

$$\begin{array}{c} certainty=\frac{H\left(0.5\right)-H\left(p_{\text{L}}\right)}{H(0.5)}=\frac{H\left(0.5\right)-H\left(p_{\text{R}}\right)}{H(0.5)} \end{array}$$ (8)

where *H*(*p*) = −(*p* log *p* + (1 − *p*)log(1 − *p*)). Thus, Eq (8) effectively measures how much information there is favoring the current heading direction.

The certainty is then used to modulate the motor update,

$$\begin{array}{c} weighted\;\;\dot{q}=certainty\times \dot{q} \end{array}$$ (9)

The incorporation of certainty is equivalent to delaying the updates of the effector without altering the nature of the optimal control (minimizing jerk). The end result is that velocity and the other kinematic changes are slowed down according to the available level of information.

### Traditional threshold model

We also implemented a more traditional threshold model [5] with five parameters: threshold (thr), drift of samples (dft), variance of samples (sig2), non-decision time (ndt), collapse rate of threshold (k). We actually tested two versions: a) one drift that functionally changed with difficulty level and b) five drifts. Results were practically similar with both versions and we decided to present the results of the simpler first version. The functional form that we used was based on a traditional formula in number cognition literature that reflects how numerical ratio affects difficulty [30],

$$\begin{array}{c} drift_{Trial_{i}}=1-erfc(\frac{abs(ratio_{i}-1)}{\sqrt{2}\times dft\times \sqrt{{\left(r_{i}\right)}^{2}+1}}) \end{array}$$ (10)

The threshold collapsed every ms as follows,

$$\begin{array}{c} threshold\left(t\right)=\frac{thr}{{\left(1+t\right)}^{k}} \end{array}$$ (11)

### Fitting

The model has three free parameters: sampling rate (*R*), confidence/acuity (*W*), and memory decay (*D*). Fitting them allows us to test the influence of these cognitive variables. Parameter values were determined with a global search of the parameter space with the DIRECT-L algorithm [42] as implemented in nloptR [43]. The algorithm minimized the sum of two mean-squared errors (MSE): 1) MSE of mean horizontal positions along the trajectory (Fig 1C), and 2) MSE of accuracy (Fig 1C). We simulated 480 trajectories per numerical ratio and computed an average simulated trajectory for the MSE. As with data, we normalized time to 101 points and use positions up to 99% of the end target to account for the gap that was not recorded (see Task and apparatus).

We did not model a button and the forces required to release it. We used the median amount of movement before participants released the button. The median value was 0.28 mm across participants. We used that reference to define RT. RT was the amount of time required to move 0.28 mm plus non-decision time. Non-decision time refers to a constant time, mainly visual and motor response delays [5]. This was set manually, after the fitting procedure, with values found in the threshold literature: 200-500 ms [7, 15].

Changes of mind were defined as correct or incorrect trajectories that penetrated 1 cm of the opposite side of the last recorded position of the index finger.

Accuracy and response times for the traditional threshold model were fitted using BADS algorithm [44] (again minimizing a function of MSE). After obtaining the best parameters we then produced positions as explained along the main text.

### Interim summary

The number comparison task (Fig 1) was modeled by combining key ideas from three influential approaches in the sensorimotor domain (Fig 2).

1. Accumulation of evidence [4]: it was assumed that subjects sampled evidence about the numerosity from each display according to Weber’s law [27, 28, 30, 45, 46]. This evidence was sampled at a rate (*R*) and it was affected by a memory decay (*D*).
2. Bayesian inference [47]: the most likely location of the correct option was inferred using the accumulated evidence and the precision of the inference was determined by an acuity parameter (*W*).
3. Optimal feedback control [18]: a minimizing jerk movement was weighted by certainty [40] computed from the probabilities of the inference.

## Results

Participants were accurate (overall accuracy = 90%) and relatively fast (mean RT between trials = 510.8 ms, s.d. = 254.5 ms). A repeated measures ANOVA revealed that accuracy (F (4,84) = 335.06, p < 0.001, $\eta_{g}^{2}=0.92$) and response times (F (4,84) = 6.34, p < 0.001, $\eta_{g}^{2}=0.08$) were sensitive to numerical ratio (Fig 1C). RTs have been previously reported to be faster when larger numbers are to the right side [48]. Nonetheless response side effects and interactions with numerical ratio were not significant (p>0.7).

Average trajectories were smooth [38, 49, 50] and the numerical distance between the arrays modulated subjects reach (Fig 1B), suggesting a fluid integration of cognitive representations to motor plans. The smoothness in Fig 1B is a result of averaging a large amount of trajectories. Still, smooth reach can be observable in single trials (random examples in S3 Fig).

**Fig 3:**
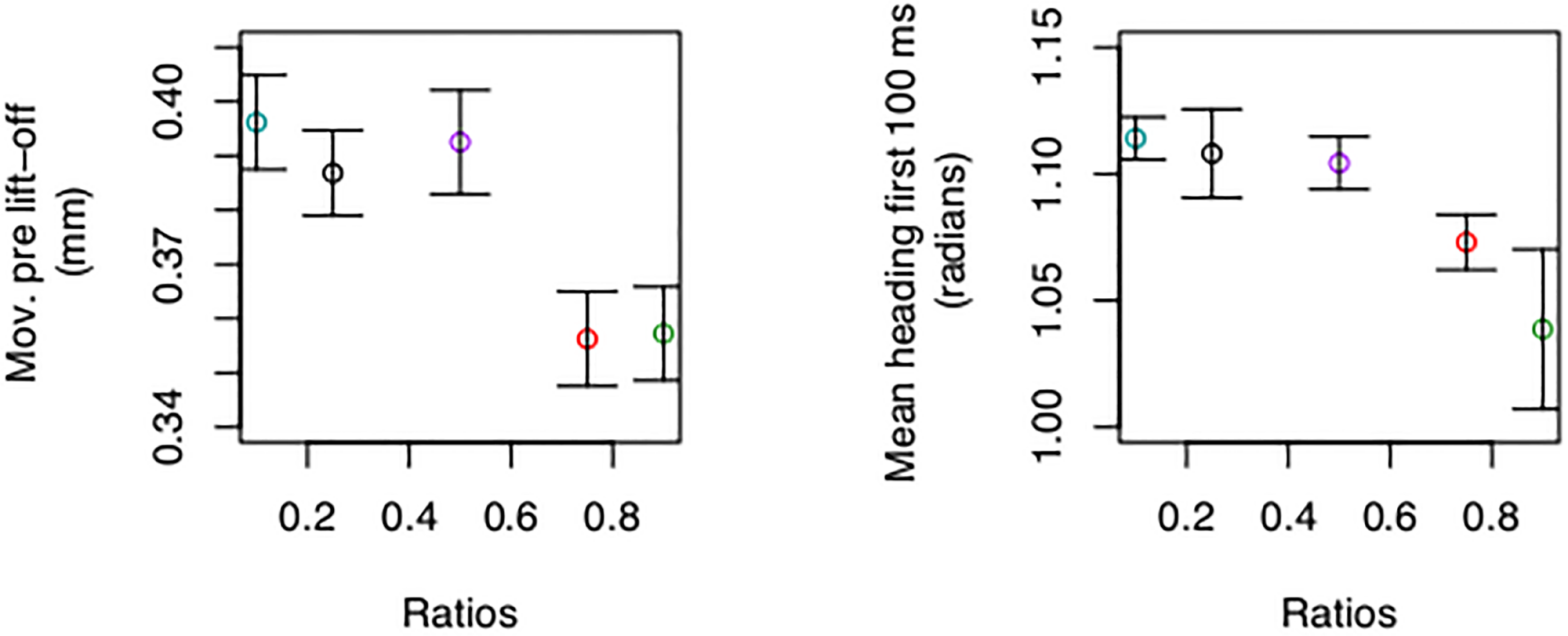
Mean micro motion before lift-off (left) and heading angle during the first 100 ms after lift-off on correct trials (right). There were early signs of modulation of movement by cognitive difficulty. Heading direction was computed as the angle of the vector formed by the infrared position and the initial position at the time of peak velocity [51]. Error bars are within s.e.m. [52].

A continuous view of decision making predicts that choice signals should be detectable at every time step of the process, not only at threshold. The mean value of displacements of the infrared marker before the button being depressed and the mean heading angle during the first 100 ms were modulated by numerical ratio (Fig 3) (micro motion: F (4,84) = 4.01, p = 0.005, $\eta_{g}^{2}=0.009$; heading angle: F (4,84) = 2.57, p = 0.043, $\eta_{g}^{2}=0.03$).

**Fig 4:**
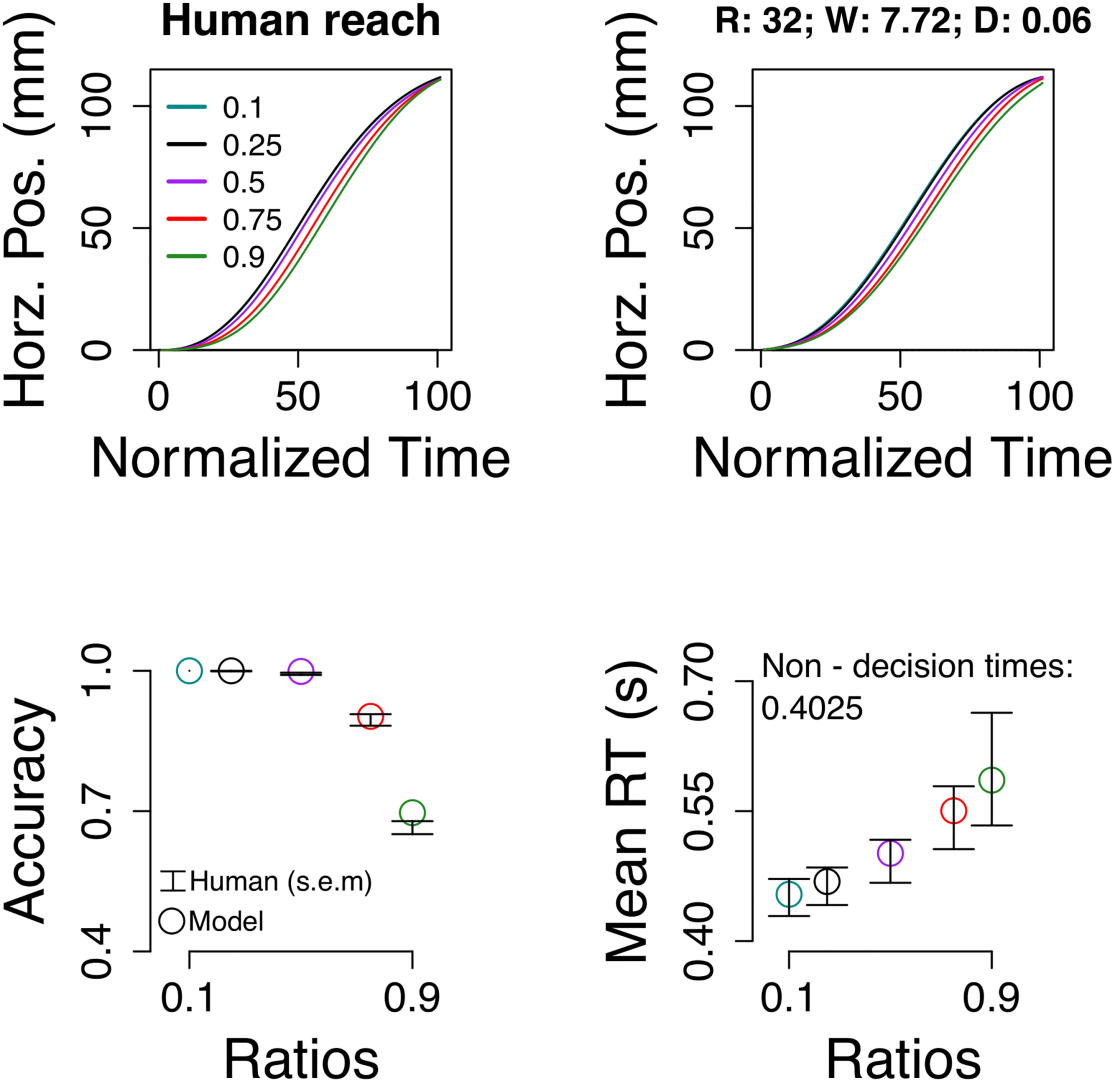
The proposed framework reproduced the sensitivity of participants to numerical ratio in reach, accuracy, and response times (for RT distributions and incorrect RTs see S7 and S8 Figs). Error bars are s.e.m.

To explain modulation of behavior in accuracy, RT, and reach, we tested a model with 3 parameters: sampling rate (R), sampling memory decay (D), and likelihood noise (W). The model reproduced the observed sensitivity to numerical ratio across the three measures (Fig 4). Such sensitivity appeared because evidence quality depends on numerical distance. For example, when comparing close numbers, say 9 dots against 10 dots, perceptual evidence is too noisy affecting the inference, delaying motor output, and producing slower response times. In general, the model accounts for classical indexes of mental processing by means of continuous statistical inference and optimal motor planning, without a decision threshold.

Perhaps because our task was memory intensive (stimulus was static and on screen for only 200 ms) the best model required strong primacy effects (D = 0.06; or, equivalently, it behaved as a noise-reduced linear accumulator). To further test how general was the process of continuous inference regardless of memory dynamics we fitted positions to the most basic model: sampling rate (R), likelihood noise (W), but no memory decay (D = 1). Even though the simulated effects were less human-like, the sensitivity to numerical ratio in reach, accuracy, and response time was still present (S5 Fig). This supports the notion that a slowly evolving motor plan that takes time to generate momentum due to statistical uncertainty is behind the observed reach gradient.

It is possible to extend the model to include decision thresholds. Samples from Eq 3 were summed up and once the accumulation got to a ± threshold motor positioning and statistical inference began (Eqs 4 and 9). Statistical inference was done with pre and post-threshold samples. The best fit model required a practically zero threshold (0.27). The reason a 0.27 threshold is practically zero is better appreciated with an example. When comparing 5 vs. 10 the mean value of evidence is 5 (see Eq 1). Moreover, assuming a Weber fraction of 0.17, the probability of an evidence value less or equal to 0.27 is roughly 0.006 (Eq 1). This means that any initial sample is enough to initiate movement i.e. no threshold is required. The alternative is to claim that 0.27 is not literally zero. We believe that this is an excessively conservative stance. To reiterate, evidence in our model is in units of numerical difference. We used numerical values in the range of 1 to 32; a 0.27 threshold is miniscule.

**Fig 5:**
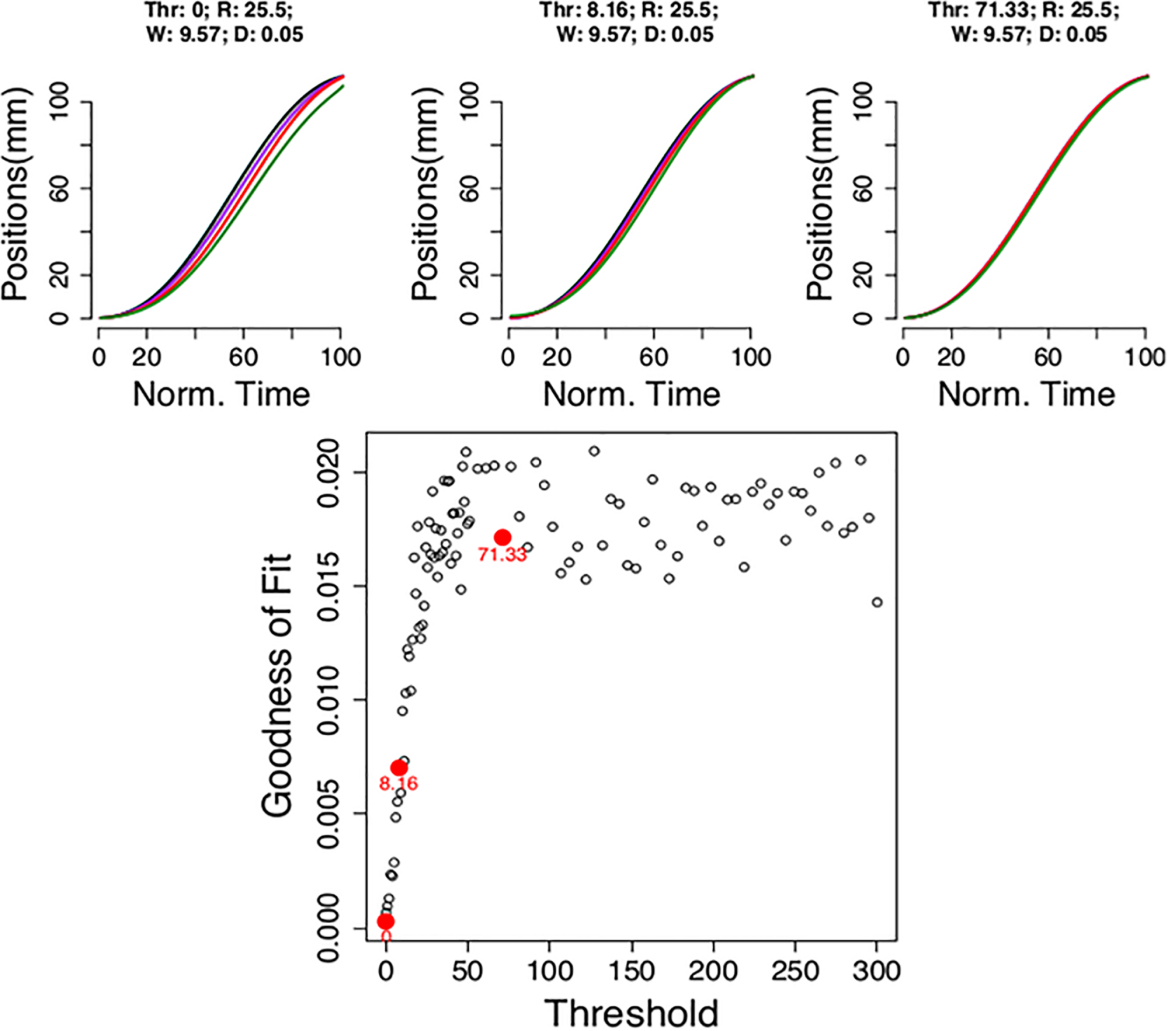
Sensitivity of the best fit model to larger thresholds. The top panels have examples of different threshold levels. Note how the reaching gradient gets weaker with larger thresholds (left to right). The bottom panel has the goodness of fit measure as a function of threshold values. Red dots are for the examples in the top panels.

A sensitivity analysis reveals the main difficulty thresholds have in explaining reaching gradients in our model (Fig 5). Sufficiently large thresholds allow for the production of enough information to confidently decide which target to hit. Thus, as thresholds increase reach becomes direct and modulations by trial difficulty disappear (Fig 5). The challenge for a threshold model is to translate evidence quality at decision to a motor plan. Our model is a plausible implementation and makes transparent one potential limitation of thresholds to explain cognitive modulation of reaching: with enough information movement should be decisive and straight to target; however this is not observed.

We also tested a more traditional threshold model with five parameters: threshold (thr), drift of samples (dft), variance of samples (sig2), non-decision time (ndt), and collapse rate of threshold (k). The general idea of traditional threshold models is that evidence is accumulated up to a decision threshold (thr). This evidence is sampled from a Gaussian distribution with mean equal to the drift (dft) and variance (sig2). The model assumes a fixed delay, called non-decision time, arising from initial perceptual encoding and motor output stages unrelated to the decision. Finally, to account for some human behavioral nuances, it has been proposed recently that the threshold should collapse as time passes by.

**Fig 6:**
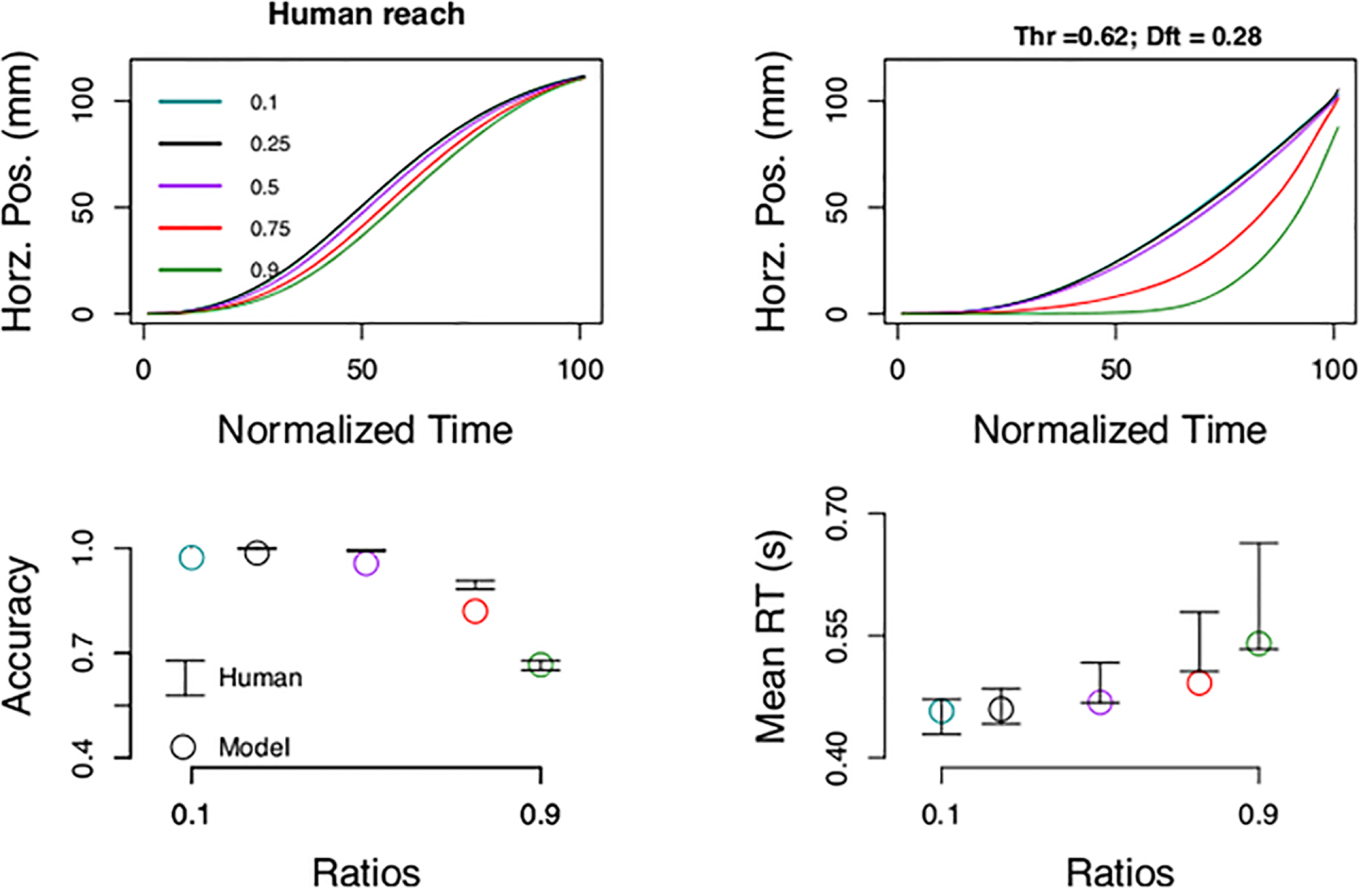
Traditional threshold model. The model reproduces human accuracy and response times (bottom panels). The mean response times for the hard ratios (red an green) are slightly faster than human participants, but the quantiles, including the median are well fitted (S11 Fig). Reaching (top right panel), however, was different from human subjects. Best fit parameters: thr = 0.62; dft = 0.28; ndt = 0.19 s; sig2 = 0.14; k = 3.56.

Traditional threshold models are excellent at replicating human discrete choice patterns and as expected the model reproduced accuracy and response times of human participants (Fig 6). However, traditional threshold models were not designed to deal with motor positioning as their objective was to explain discrete choice [5]. To generate motor positions, we computed the posterior probability of being correct as follows,

$$\begin{array}{c} p\left(Corr|samples\right)=\frac{p(samples|Corr)p(Corr)}{p(samples|Corr)p(Corr)+p(samples|Incorr)p(Incorr)} \end{array}$$ (12)

the prior of being correct or incorrect is p(Corr) = p(Incorr) = 0.5, the likelihoods p(samples|Corr) and p(samples|Incorr) were assumed Gaussian i.e. ∼ Normal(dft, sig2) and ∼ Normal(-dft, sig2), respectively. It has been proposed before that a proxy of confidence is to compute a likelihood ratio between both hypotheses and place a new threshold on that metric [53]. But the question remains on how to transform that likelihood ratio to motion. Instead, to compute certainty (Eq 8), we used the posterior in Eq 12 because it is already in probability space, and updated positions with Eq 9. We further assumed that sampling continued after hitting the threshold.

It is important to highlight that this model differs from our previous threshold model in that it has a collapsing threshold. Also, the parameters of the Gaussian evidence: the drift or mean is related to cognitive difficulty via Eq 10 and the variance is not connected to cognitive difficulty. And finally, in the posterior: one posterior is based on two discrete hypothesis (Eq 12) and the other on a continuous hypothesis space, namely the estimate of the numerical difference (Eq 7).

The traditional threshold model replicated discrete choice, namely accuracy and response times, but it could not displace the index finger to the target (Fig 6). We tried to include a second post-lift off threshold for changes of mind and a post-lift off deadline to stop accumulation [15] and select the target with highest posterior. However, it was not possible to replicate the position gradient even with the addition of these two parameters (S10 Fig). We note that this result depends on the posterior used (Eq 12) and the general setup.

Thresholds can explain discrete choice (Fig 6). Continuous output, however, impose a challenge on the framework that requires non-trivial extensions. For instance adding post-lift off thresholds, time limits on accumulation, formalization of confidence metrics, and their interaction with motor plans. We could not strike the correct balance so that a single or multiple stage discrete threshold model could explain the data (but in principle there should exist an appropriate one). A continuous inference scheme, on the other hand, was successful in approximating human choice patterns.

### Changes of mind

**Fig 7:**
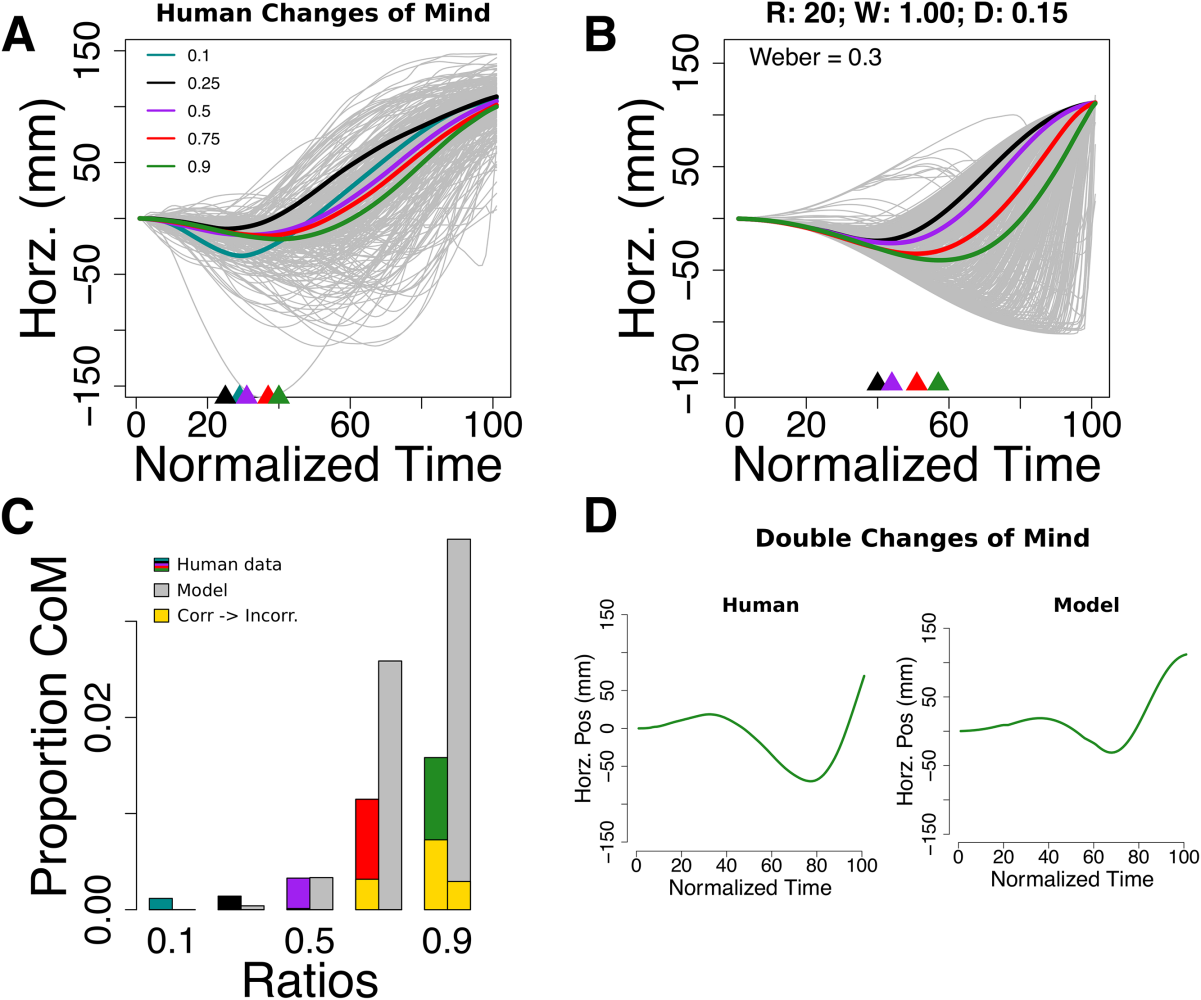
Changes of mind without post-lift off thresholds. (**A**) Changes of mind in human participants. Thick color lines are averages colored by numerical ratio. Arrows on the x-axis are a mean proxy of where the change of mind first appeared (the x coordinates of the minimum points of the thick lines). (**B**) Changes of mind produced by a parametrization of the model. (**C**) They increased with cognitive difficulty, both in the model and human data. The minority of changes of mind were from correct to incorrect (gold). (**D**) Examples of double changes of mind in the model and human data.

Changes of mind are deviations of the motor effector toward a target different from the initial selection. They are of interest because they confirm the presence of online processing during reach. Even though the model and the static nature of the stimulus were not designed to probe changes of mind, the model was able to produce them (Fig 7D).

Also, we observed more than one change of mind in some trials (Fig 7D). In the model, changes of mind are the result of continuous statistical fluctuations. This means that multiple changes of mind are possible without additional assumptions (Fig 7D; they are highly scarce in human data and the model (<0.3%).

Thirdly, a manually-tuned parametrization of the model replicates known characteristics of changes of mind reported in previous literature [15]: 1) highly scarce and more prevalent in hard trials (Fig 7C); 2) they tend to fix an error i.e. they go from incorrect to correct (Fig 7C); and 3) most of them appear relatively far from the target depending in trial difficulty (see arrows in Fig 7A and 7B). Therefore, the model in Fig 7 represents a plausible average cognitive state present when changes of mind occurred. This was obtained with a manually tuned model because fitting the model with the global method by adding changes of mind (see Methods) produced a mildly informative compromise between positions, accuracy, and changes of mind. This may only testify about the difficulty to fit 3 dependent variables at once. More relevant is that the manually-tuned parameter values were insightful about changes of mind as continuous in nature. Note, however, that the parameter values obtained by fitting positions and accuracy (Fig 4) produced too few changes of mind (< 1%). Subjects’ cognitive parameters may have fluctuated to produce the rate of observed changes of mind (∼3%). The details of such fluctuation are beyond this work but could be caused by drops in attention or fatigue, leading to a higher Weber fraction or an altered likelihood noise.

Importantly, changes of mind can occur in the proposed continuous regime and comply with features found in previous behavioral work [15]. For example, in the model the reason that changes of mind tend to be prevalent in hard trials and correct an error is that an initial stream of incorrect evidence is more likely to occur when numbers are close (Eq 1) but as more samples arrive, and because there is a correct answer, the initial stream of incorrect evidence is overrun and the inference changes. Similarly, the point where a change of mind occurs along the reaching trajectory depends on numerical distance (see colored triangles in Fig 7A and 7B) because difficult comparisons generate low quality information taking longer to overrun an initial stream of incorrect evidence.

### Weber fractions

**Fig 8:**
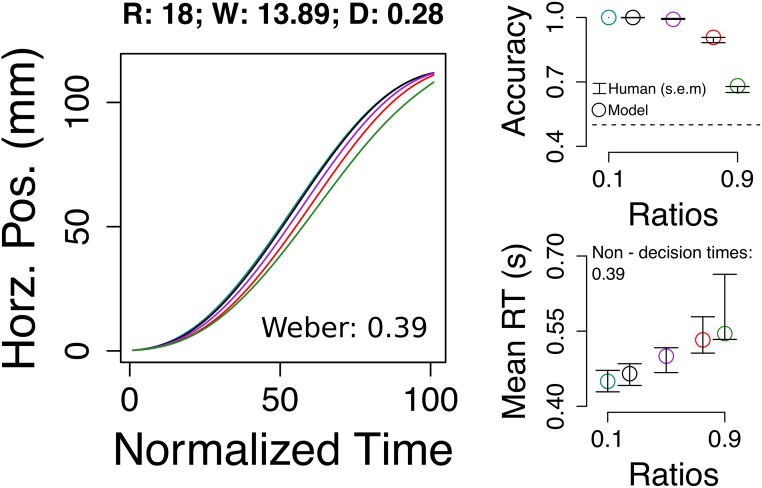
Weber fraction estimation from dynamic (reach) and discrete (accuracy) dependent variables. In all the previous figures the Weber fraction was a fixed parameter obtained from subjects’ accuracy, as traditionally done in most psychophysics papers of numerosity. In this figure, we fitted a model with the Weber fraction as a free parameter. This means that the parameter is obtained both from dynamic reach and discrete accuracy. By including reach, we obtained a higher Weber (0.17 vs 0.39) i.e. a noisier perceptual system that reproduces the same accuracy and reach. Error bars are s.e.m.

So far we used the Weber fraction *ω* estimated from accuracy, following standard practices in number cognition literature (Eq 2). With the model it is possible to estimate it using subjects’ positions *and* accuracy (see Methods). That is, we add *ω* as a free parameter in the fitting procedure. We found parameter values that reproduced sensitivity to numerical ratio in reach, accuracy, and response time (Fig 8). The obtained Weber fraction *ω* was higher than the one obtained from accuracy alone (0.17 vs 0.39). A perceptual system that is noisier, as revealed by a higher Weber fraction, reproduces accuracy. In other words, even though it is possible to estimate a low Weber fraction from accuracy the underlying uncertainty, as revealed by reaching, is not captured by just using percentage of correct answers. However, it is worth to highlight that the larger Weber fraction may also be a reflection of a strong memory effect in sampling (D = 0.06). Future experiments could try to separate memory effects from purely perceptual noise.

## Discussion

We presented a model that translated a high level cognitive representation, number, into dynamic action. Our threshold-free model accounted for the subtleties of human numerical decision-making, including accuracy, response time, the spatial layout of reach trajectories, and changes of mind.

### Number cognition

There is a considerable amount of work exploring number representations and their effects on movement plans [17, 54–56]. The novelty of our work is a formalization of how psychophysical representations of number may be projected to reach. Two relevant theoretical insights are worth noting. First, the model never directly projected the details of the numerical representation. Previous work has proposed otherwise and suggests that the motor system forces logarithmic scales or numerical formats in how the motor effector, i.e. index finger, is moved in physical space [25, 54, 57]. In our model the displacement of the index finger is not forced to follow precise aspects of the number representation; it is the state of the inference and confidence that is being reflected along the reaching trajectory (see a similar discussion in [56]). In a broader sense, movement may only be a window to a domain-general process, such as confidence, rather than domain-specific aspects of cognition (log. scaling of mental space).

Second, the model allowed us to estimate Weber fractions using both accuracy and movement patterns. We found a larger Weber fraction than the one estimated from accuracy alone (0.39 vs 0.17, respectively). This suggests that human numerical sensitivity may be misestimated if confidence and decision dynamics are not taken into account. This has direct implications for math education given the efforts to connect perceptual numerical estimation, number metacognition, and formal math learning [31, 58, 59]. By bringing forth the idea that numerical estimation may be affected by confidence or other decision parameters, such as memory in sampling, it may be possible to explain why some researchers find positive and others negative results regarding the importance of approximate numerical estimation in formal/symbolic mathematics; an argument also extended in [11]. In brief, the observed relation between the approximate number system and mathematics may be mediated by aspects unrelated to numerical Weber ratios (e.g. perhaps the quality of the inference varies between students, as indexed by the parameter W in our model).

### Thresholds and action

In most domains decision thresholds are useful for describing a large number of brain and behavioral responses [4, 5]. However, our results show that in some very simple computational systems, threshold-like behavior may arise as essentially an epiphenomenon: behavior may be effectively delayed due through uncertainty over motor plans, not inherent cognitive or neural mechanisms that set bounds for action. This suggests that in some domains thresholds may be replaceable with other rational processes.

Thresholds are a dominant framework in the field. However, there are prior reports of their failure to capture some aspects of behavior. In a recent neurophysiology paper, one of the monkeys failed to show neural signatures of a threshold when selecting one of the options [60]. The monkey’s neurons behaved as if they were not accumulating evidence, even though the accuracy of the monkey in the task was high. In another study testing for sources of noise in the accumulation process, it was found that subjects (rats and humans) were best fit by models with infinite thresholds [35]—bounds were not necessary. In work that used dynamical systems to model behavior it was found that very low thresholds were required to produce changes of mind [61], and that fast response times and high spike rates were not correlated as predicted by a rise-to-threshold theory [62]. Finally, neural responses in canonical accumulation areas can be explained by discrete steps rather than ramping accumulation [63], plausible indicating the production of discrete samples instead of a rise-to-threshold.

Our study complements these findings and strongly suggests that once motor models are combined with statistical inference models, threshold-like behavior may emerge as a consequence of motor planning. This work therefore points towards models which make explicit the interrelation of decision and action, and show how interesting behavior may emerge from the confluence of simple processes in inference, choice, and movement.

An interesting result was that micro movements before button release were modulated by numerical ratio, suggesting information processing early on. There are precedents on this idea. Micro eye motions during fixation have been connected to active information processing that transforms seemingly static visual space to temporal signals interpretable by the retina [64]. Studying early signals becomes important in a continuous decision framework but can also inform on whether choice is discrete or not.

Decision making is highly likely to be a mixture of discrete and continuous mechanisms. The results herein are not directly undermining the whole idea behind thresholds. In fact, it should be possible to come up with a discrete scheme that explains the observed human behavior in our task, perhaps by adding multiple post-lift off thresholds. Rather, we think the main insight is that decisions can be placed in a continuous framework, even for simple static comparison tasks. It would be interesting to extend this to classic dynamic contexts such as the random dot motion task, usually modeled with a discrete framework.

### Choice error and movement uncertainty

The task design (stimulus on-screen for only 200 ms) and model results (D = 0.06) suggest that subjects gathered evidence from memory or, equivalently, that they accumulate non-stochastically and linearly [10]. In general, the literature suggests that both of them are plausible [10, 32]. However, it is important to clarify that this implicates that for the model the source of error made by subjects is mostly due to a bias in the mean of the internal memory representation. The initial samples strongly bias subjects and when wrong they produce mistakes. That is, the source of error is driven mostly by a bias in the perceived mean of the internal representation, rather than its variance.

Another intriguing aspect of our results is that it is unclear how to move when there is ambiguous evidence. In our task, ambiguous information appears in trials with equal number in both sides (S6 Fig). A simple average of parallel motor plans to each option will make the movement land in between the options (e.g. [65]). Similarly, weighting by amount of information, as we did here, will fail to fully drive the index finger to the target (S6A Fig). Dealing with uncertainty gridlocks is a difficult challenge for future decision-making models that try to account for movement patterns, accuracy, and response times in 2AFC. Still, we present a possible solution using a satisficing strategy in the supplemental material (S6 Fig).

A signature of uncertainty is the presence of changes of mind [15]. We propose that they result from an early incorrect inference due to initial numerical evidence favoring the non-selected target. Also, because the model produced changes of mind with slightly different parameters than the best fitted ones, they may require fluctuations in cognitive parameters; perhaps brought about by external variables, such as drop in attention or fatigue. More interesting is that changes of mind in number comparisons can be framed as continuous events rather than the arrival to a discrete post lift-off threshold [15].

## Supporting information

S1 Fig Effects of the memory parameter *D*. Each row has the effects of different memory parameters in an example series. Column 1: orange trace is the original sample series; black trace is the EMA series. Column 2: illustrates how the weight of each previous sample decays as more samples arrive.
(PDF)

S2 Fig Left (+) and right (-) trajectories. Upper and lower heat bars are pointwise significance effects of numerical ratio of a functional ANOVA. Shading is 2 s.e.m.
(PDF)

S3 Fig Examples of randomly picked trajectories. Rows organized by numerical ratio (0.1, 0.25, 0.5, 0.75, 0.9) and colored as in Fig 1B.
(PDF)

S4 Fig Movement modulation in different axes. Number ratio effects were stronger in the horizontal axis. Shading is 2 s.e.m.
(PDF)

S5 Fig No memory decay. A model without memory decay is also sensitive to numerical ratio in reach, accuracy, and response time.
(PDF)

S6 FigModel and participants in trials with equal number.(**A**) The best fit model in Fig 4 favors one side even in these trials (blue trace). This happens thanks to the limited number of stochastic samples in a brief reach lasting 600 ms on average. (**B**) In trials with equal number human participants fully arrive to target, suggesting a type of satisficing strategy that settles to the closest target. (**C, D**) A modification of the model that implements a satisficing strategy unrelated to the inference successfully arrives to target in all numerical ratios (**C**), and replicates accuracy and RT patterns (**D**). Dashed line in accuracy plot is chance. In the model motor plans were modulated by certainty. This seems to predict that when both options are identical, say 10 dots to the left and right, subjects will end up at exactly midway between the targets due to maximal uncertainty. Contrary to this assumption, in a movement that is time and space constrained, the amount of stochastic evidence is limited and one side would be favored. We tested the model using the parameter values in Fig 4 and simulated trials with equal number on both sides (number ratio = 1). Interestingly, in these trials average reach is dominantly tilted to one side (S6 Fig, A). To fully arrive to target subjects could produce a top-down signal and satisfy. For example, as the index finger gets closer to the computer screen they settle towards the closest target. Satisficing strategies are well known in decision-making literature [66] and can be implemented in the model with a simple extension to Eq (9), $$\begin{array}{c} weighted\;\;\dot{q}=certainty^{distance}\times \dot{q} \end{array}$$(13)
*distance* is the normalized depth distance to the screen (0 screen arrival and 1 start button). As the finger approaches the screen the pull exerted by certainty becomes weaker and the movement is mostly driven by the kinematic update $\dot{q}$ (e.g. at screen arrival: *certainty*^0^ = 1). Physical distance is not changing certainty levels, just how relevant it is for updating motor positions. To observe what human participants do and test the extended model, we ran a new experiment with trials that had equal number of dots on each side (tested numerical ratios: 0.25, 0.5, 0.75, 0.9, 1; n = 22, mean age = 19.91 years, s.d. = 1.80, Weber fraction = 0.15). Subjects and the model got to target in trials with equal number (S6 Fig, B,C). The model was also successful in reproducing sensitivity to numerical ratio in accuracy and response times (S6 Fig, D). In this experiment, though, participants’ RT flattened out in the hardest numerical ratios (0.9 and 1). It is unclear why but recently it was demonstrated that motor initiation in humans is usually delayed ∼80 ms and under volitional control [67]. Movement initiation is part of a non-decision time which means that the assumption we made of being fixed is wrong (but also found in all evidence-to-bound models). These participants may have reduced the delay in movement initiation for the hardest trials to optimize time spent in the task. More important is that the flattening out of RT and satisficing strategies do not contradict the general argument that subjects modulate their movements based on a rational inference.(PDF)

S7 Fig Distribution of response times. The model reproduces fairly well response times (quantiles 0.25, 0.5, and 0.75) but the distribution of RT for subjects is noisier (see extreme quantiles 0.1 and 0.9). We argue that such additional noise may be in part explained by muscle forces or flight time estimations which were not explicitly included in the model and could have been affected by certainty levels. Ratio 0.1: cyan; 0.25: black; 0.5: purple; 0.75: red; 0.9: green.
(PDF)

S8 FigResponse times in incorrect trials.Similarly to human participants, the model was slower in incorrect trials. Ratio 0.1: cyan; 0.25: black; 0.5: purple; 0.75: red; 0.9: green. Lower right panel is overall mean across ratios (F(1,21) = 6.10, p = 0.02, $\eta_{g}^{2}=0.05$). Error bars are s.e.m.(PDF)

S9 Fig Index motion by performance. **Mean reach in correct and incorrect trials of humans (left panel) and the best fit model (right panel)**. Incorrect trials are more medial in the model than in humans. This is similar to what happens in trials with the same numerosity on each side (S6 Fig): Evidence quality is weak and hinders arrival to the target. We reasoned that humans may be using a top-down signal to hit the target. See a plausible implementation of such top-down signal in S6 Fig i.e. distance to the screen is used as a cue to stop relying in evidence and instead try to arrive to target (Eq 10).
(PDF)

S10 Fig Change of mind threshold and movement. Adding a second post lift-off threshold and a deadline to stop accumulation, as in [15] did not improve motor positioning of the traditional threshold model. On average, they collapsed to a similar trajectory (top right). After the second threshold the model makes a definitive commitment and on average the finger goes directly to target. The fitting procedure could not find a better solution. Best parameters: thr: 0.62, 2nd thr: 1.02, deadline: 51 ms; dft: 0.28; ndt: 0.19 s; sig2: 0.14; k: 3.56.
(PDF)

S11 Fig Response time quantiles for the traditional threshold model. Traditional threshold models are excellent at reproducing response times.
(PDF)
