# Supplementary figures and images for: A threshold-free model of numerosity comparisons
Source: PLoS One. 2018 Apr 5;13(4):e0195188. doi: 10.1371/journal.pone.0195188 (PMC5886529; doi:10.1371/journal.pone.0195188)

**Mem. parameter = 0.05**

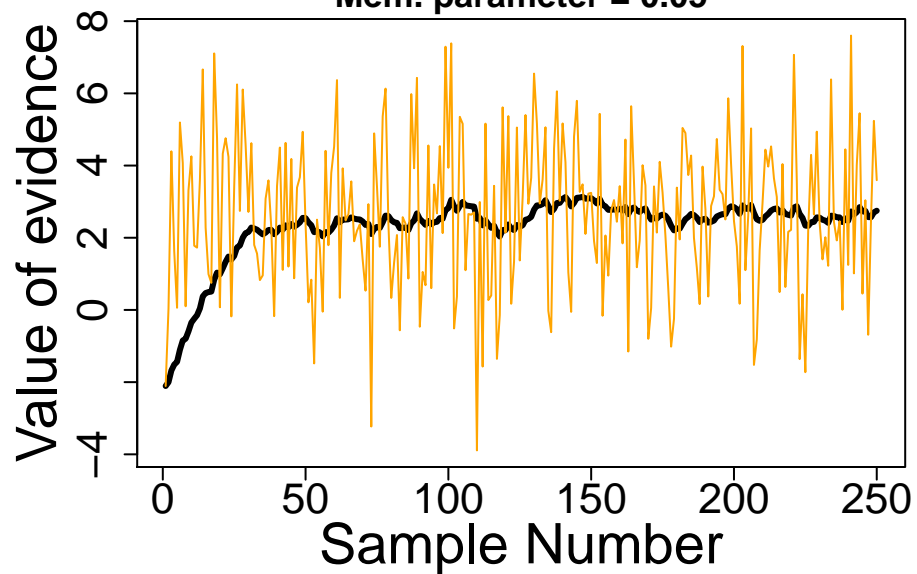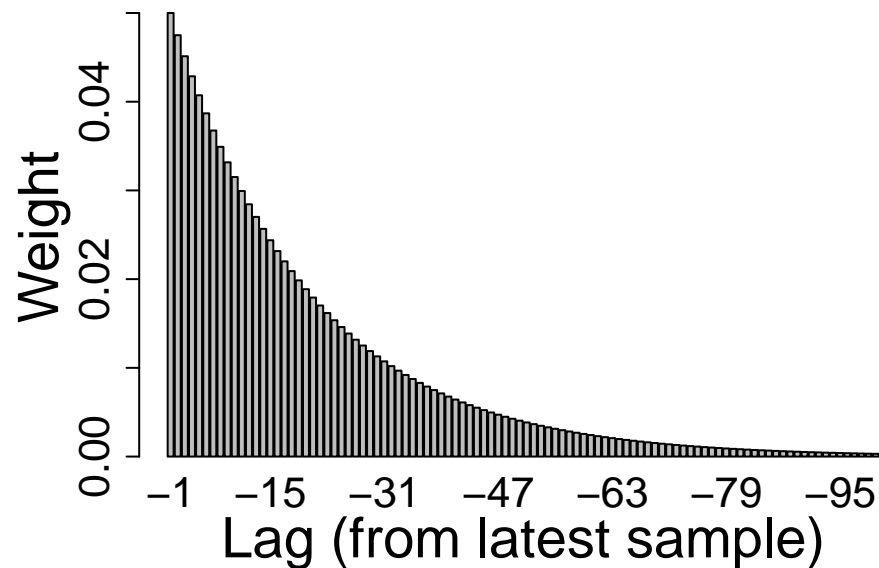

**Mem. parameter = 0.50**

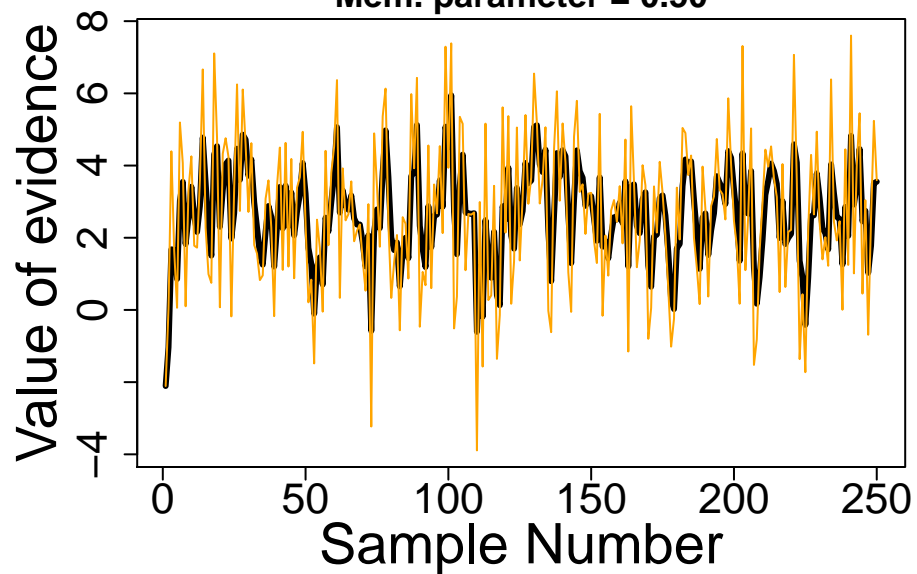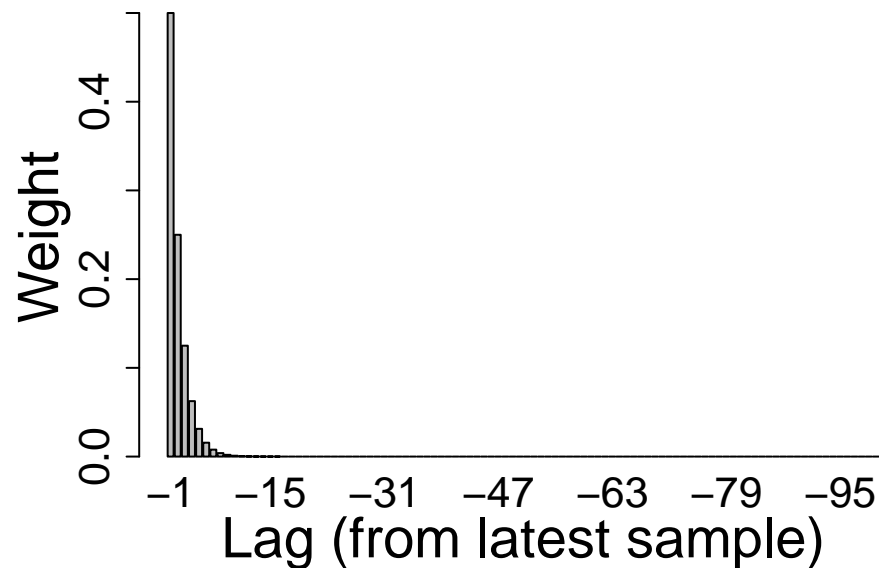

**Mem. parameter = 1.00**

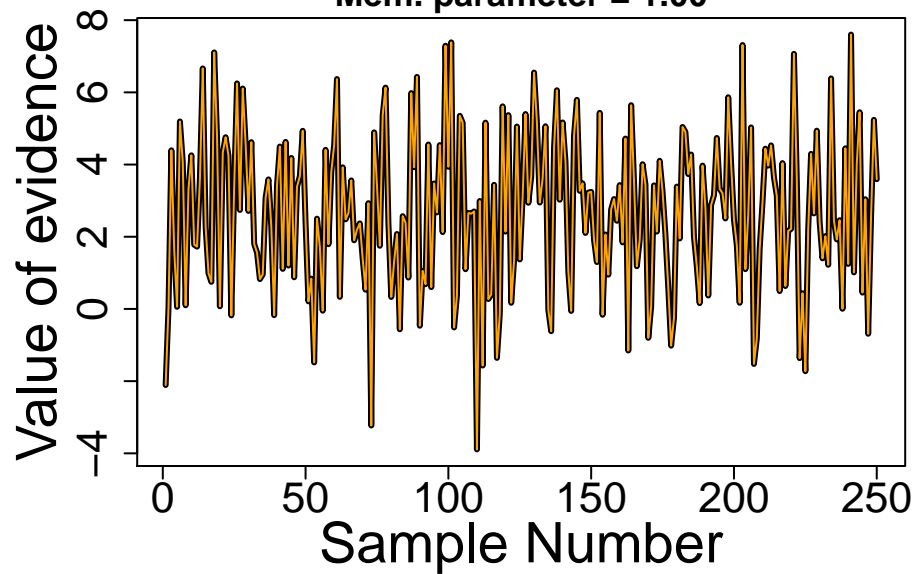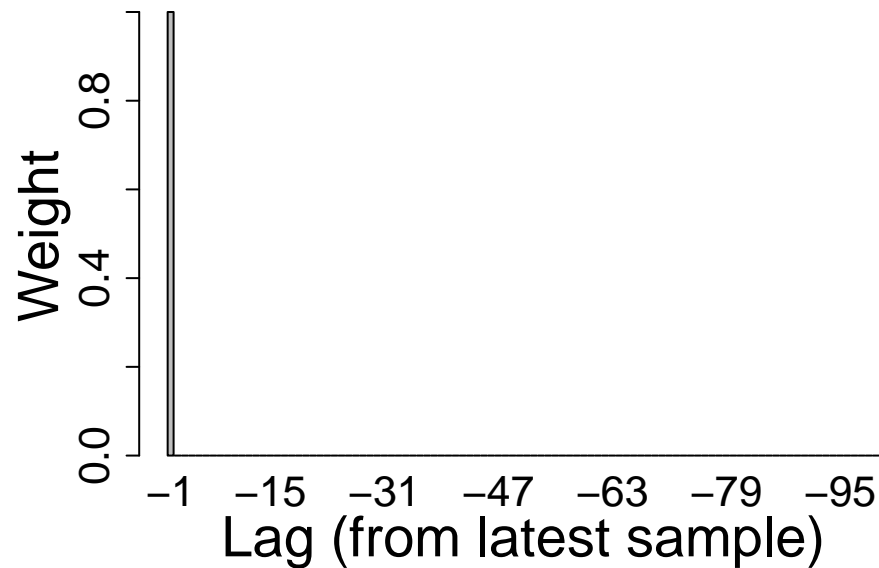

Supplement: S1 Fig — Each row has the effects of different memory parameters in an example series. Column 1: orange trace is the original sample series; black trace is the EMA series. Column 2: illustrates how the weight of each previous sample decays as more samples arrive. (PDF) [file pone.0195188.s001.pdf]

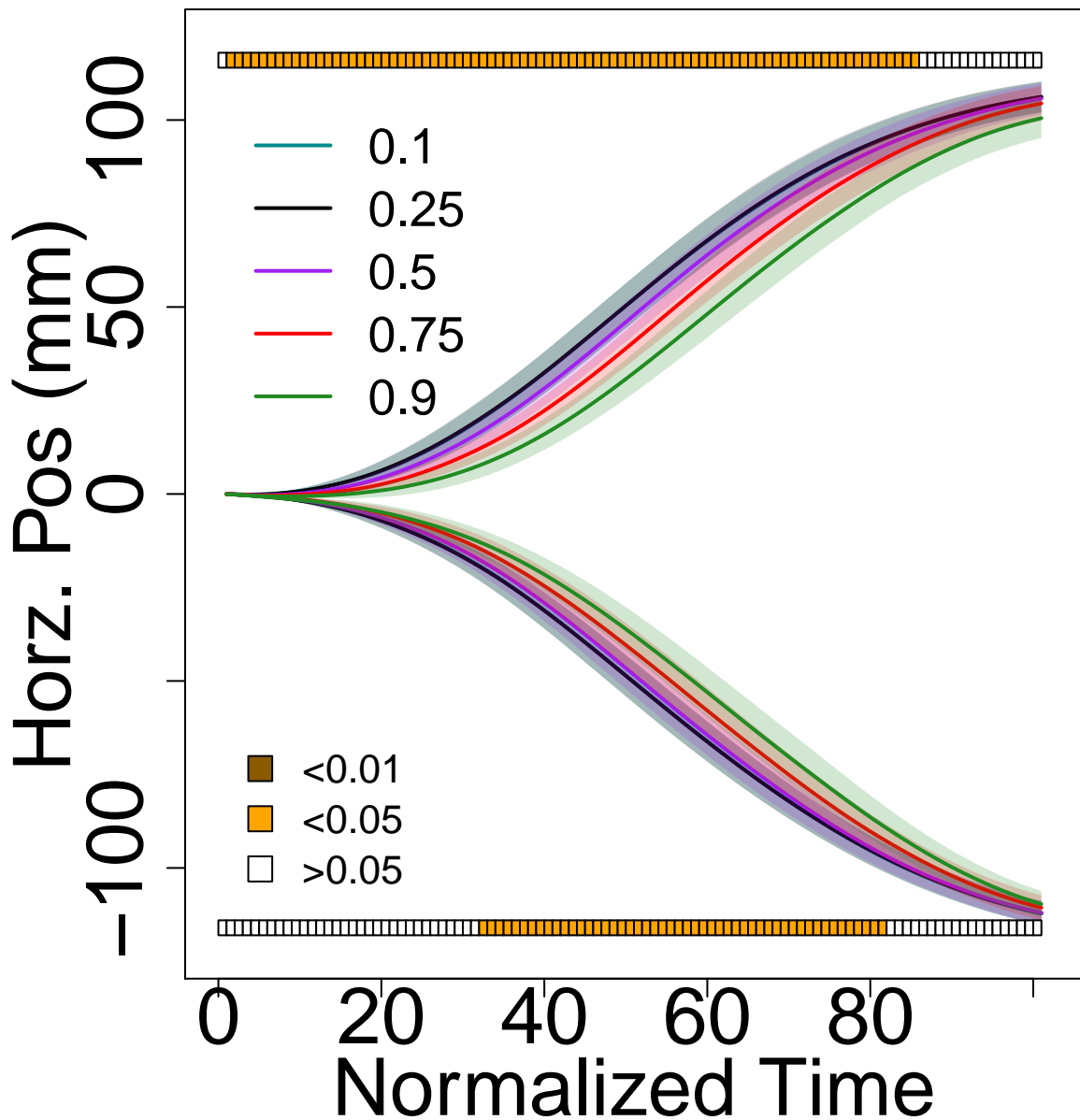

Supplement: S2 Fig — Upper and lower heat bars are pointwise significance effects of numerical ratio of a functional ANOVA. Shading is 2 s.e.m. (PDF) [file pone.0195188.s002.pdf]

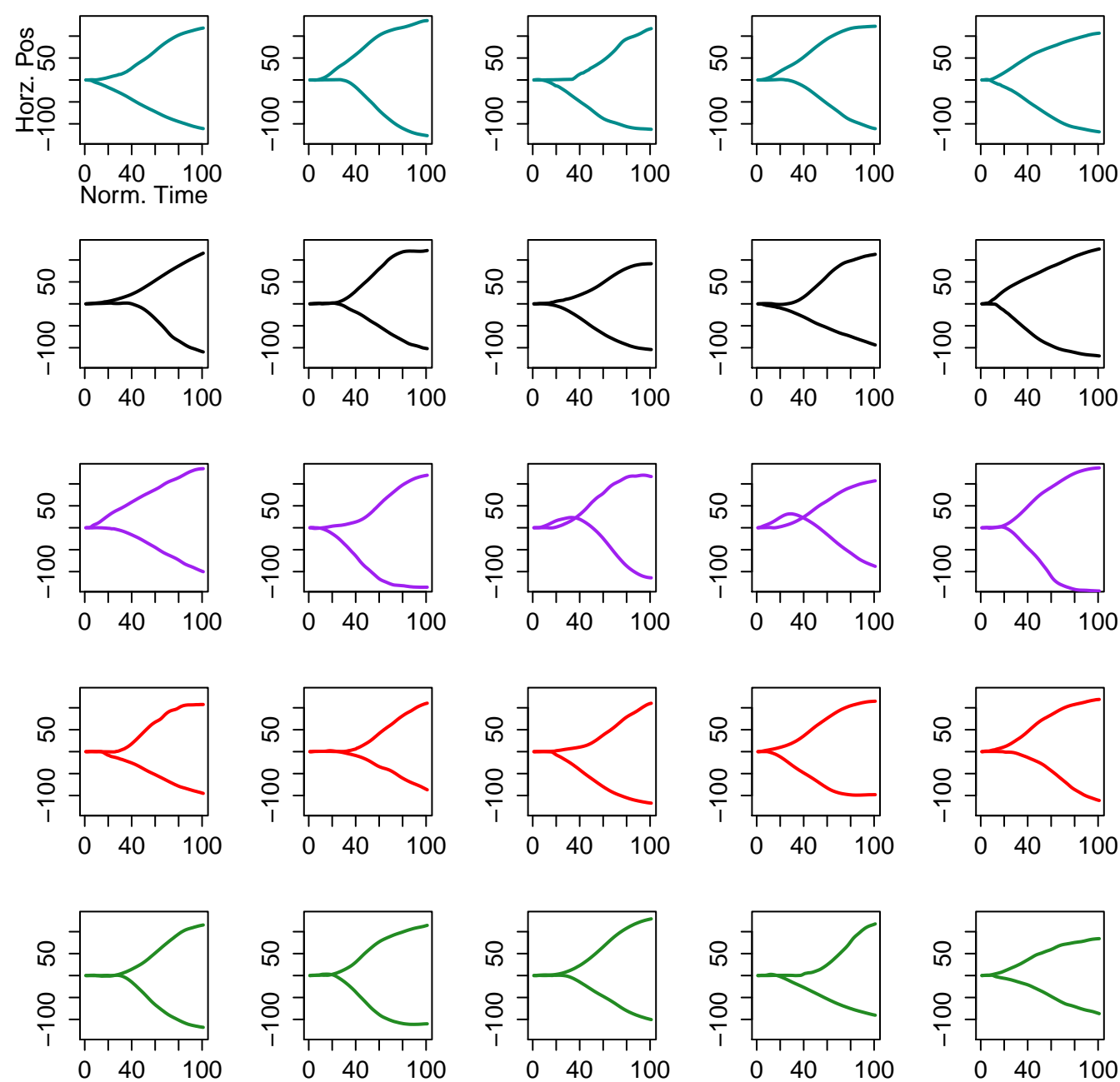

Supplement: S3 Fig — Rows organized by numerical ratio (0.1, 0.25, 0.5, 0.75, 0.9) and colored as in Fig 1B. (PDF) [file pone.0195188.s003.pdf]

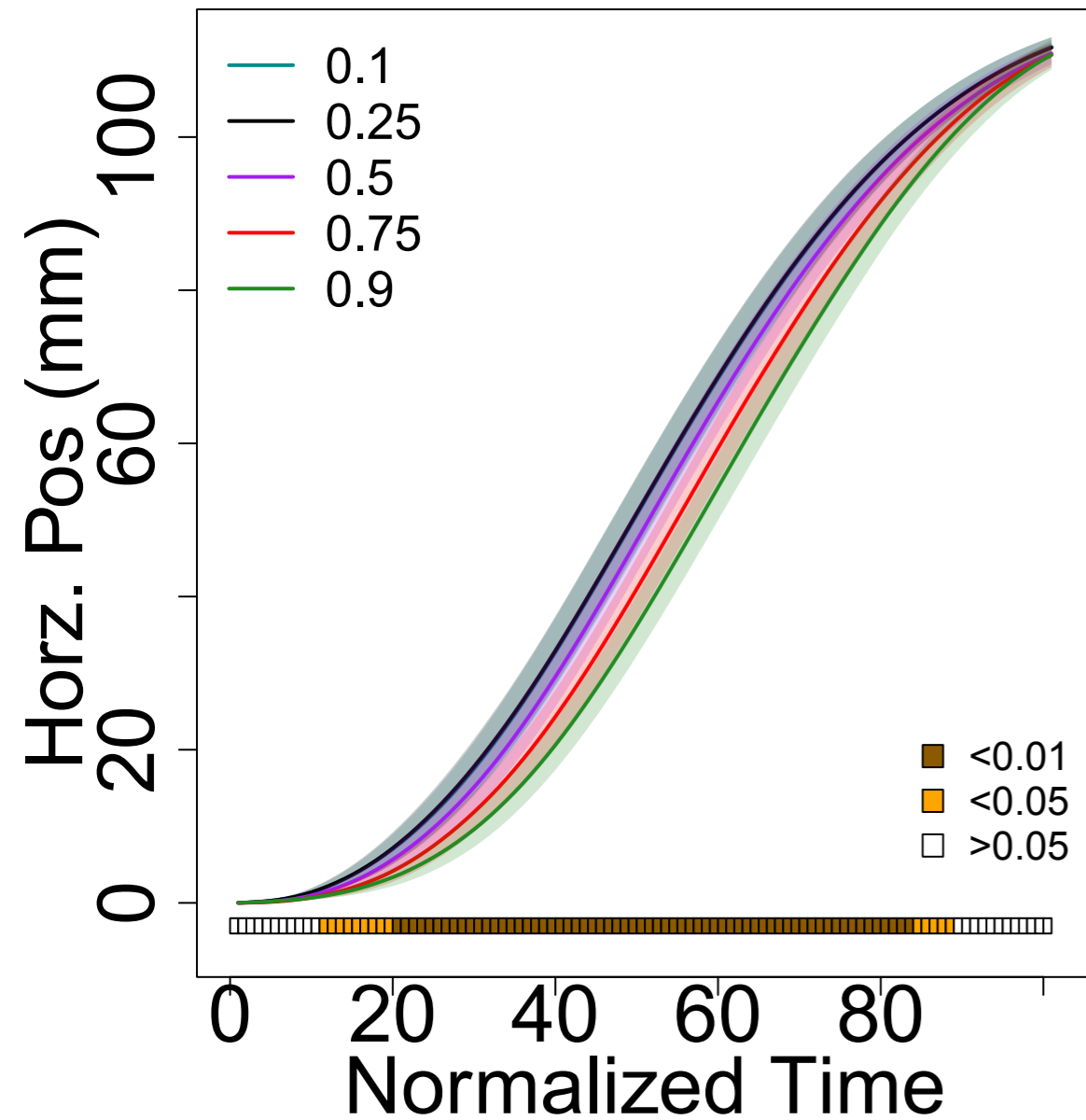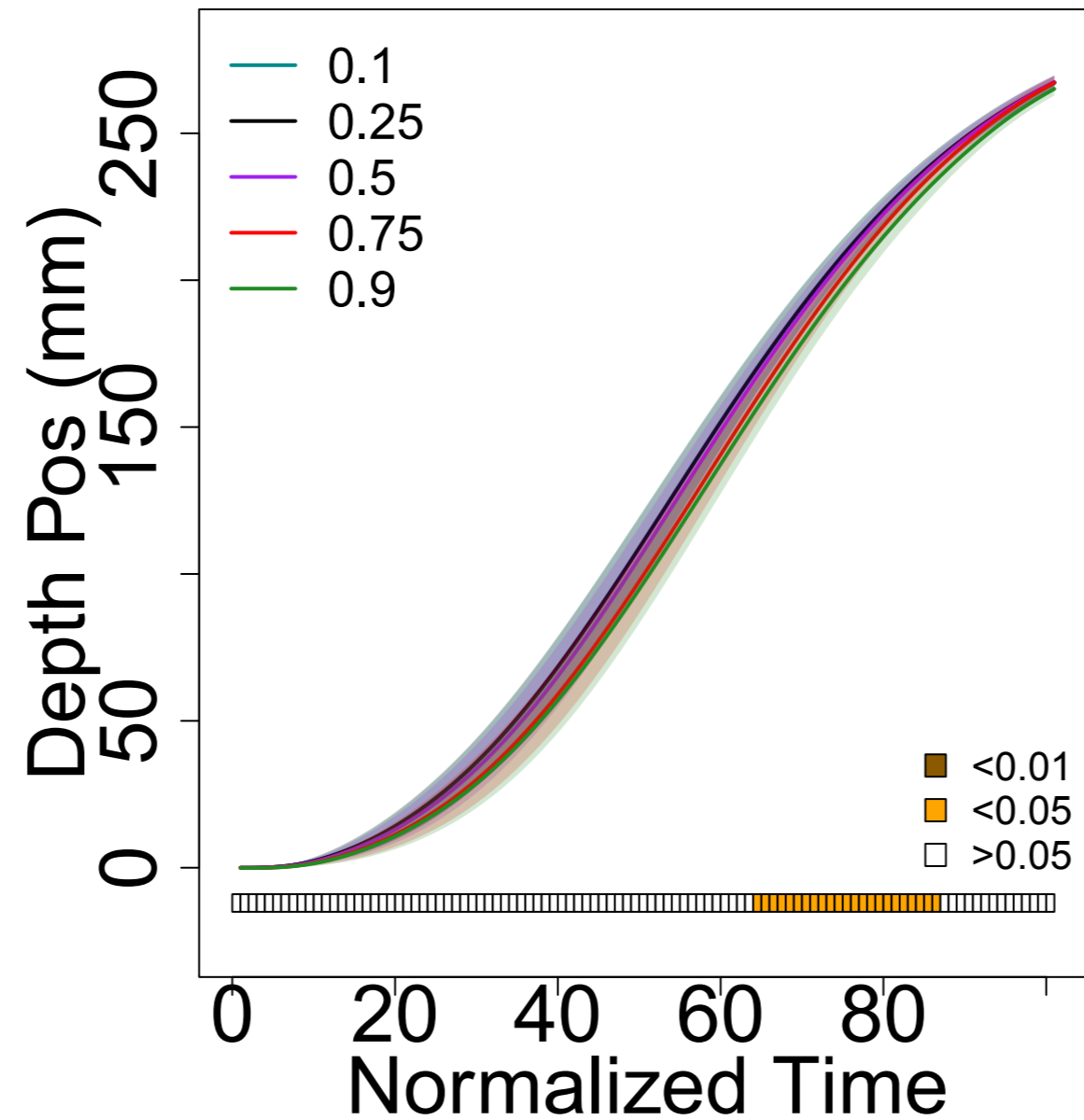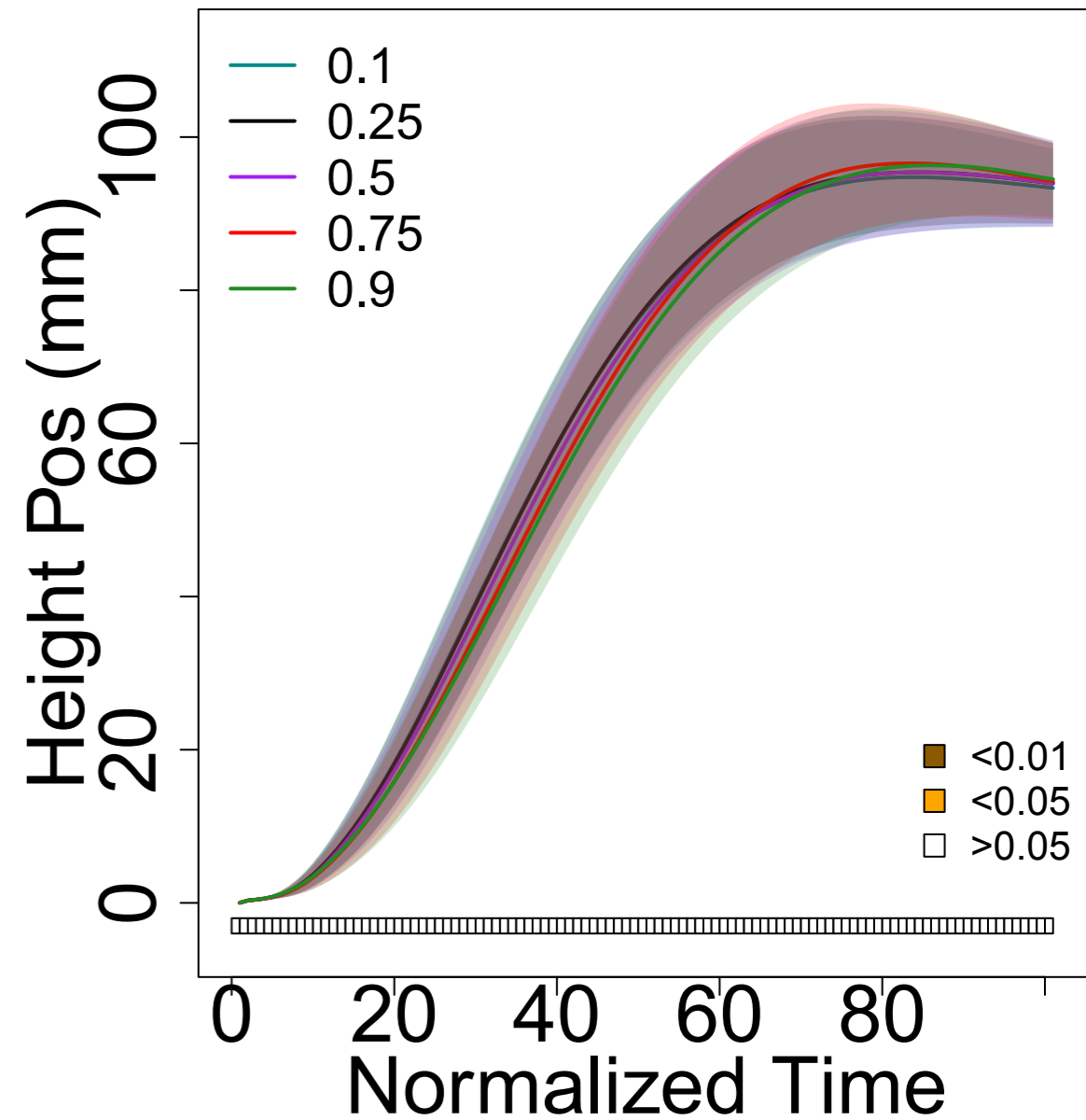

Supplement: S4 Fig — Number ratio effects were stronger in the horizontal axis. Shading is 2 s.e.m. (PDF) [file pone.0195188.s004.pdf]

**Human reach**

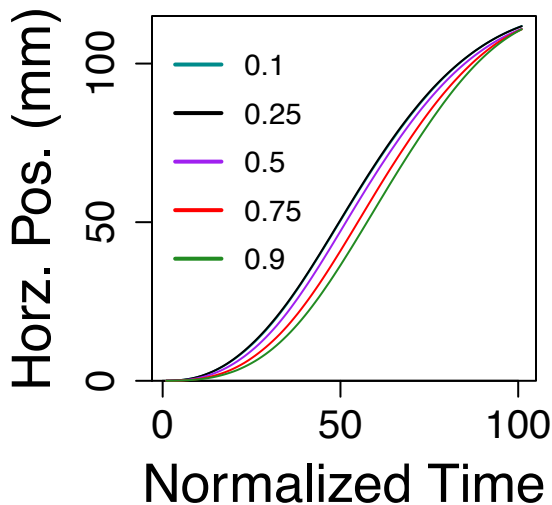

**R: 74; W: 2.57; D: 1.00**

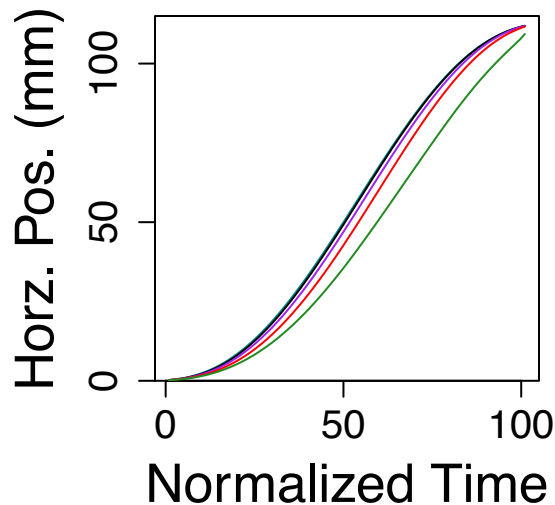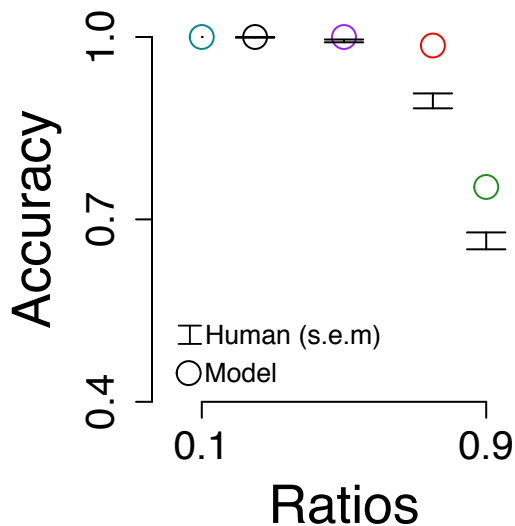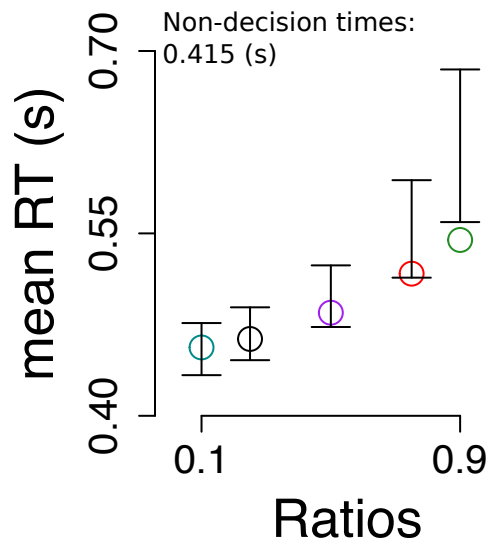

Supplement: S5 Fig — A model without memory decay is also sensitive to numerical ratio in reach, accuracy, and response time. (PDF) [file pone.0195188.s005.pdf]

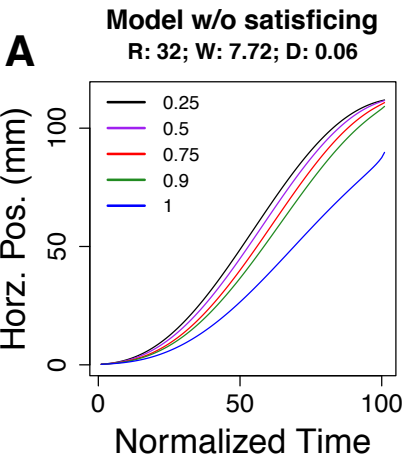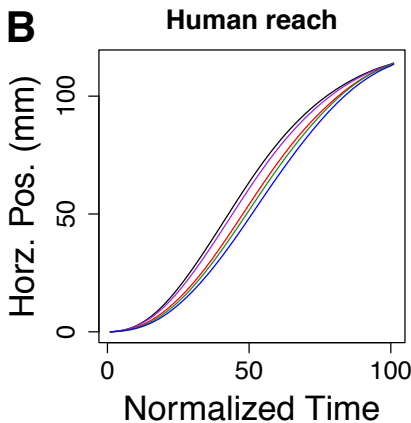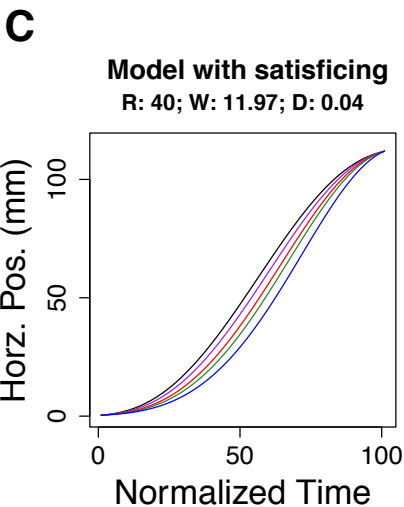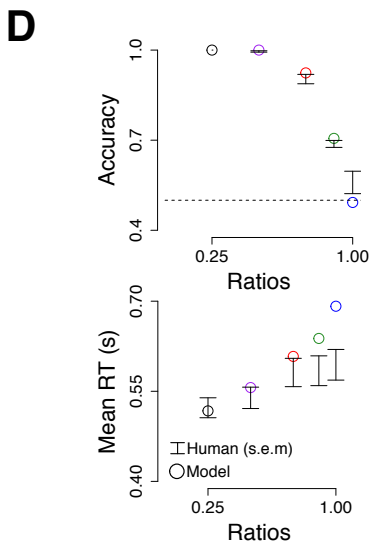

Supplement: S6 Fig — (A) The best fit model in Fig 4 favors one side even in these trials (blue trace). This happens thanks to the limited number of stochastic samples in a brief reach lasting 600 ms on average. (B) In trials with equal number human participants fully arrive to target, suggesting a type of satisficing strategy that settles to the closest target. (C, D) A modification of the model that implements a satisficing strategy unrelated to the inference successfully arrives to target in all numerical ratios (C), and replicates accuracy and RT patterns (D). Dashed line in accuracy plot is chance. In the model motor plans were modulated by certainty. This seems to predict that when both options are identical, say 10 dots to the left and right, subjects will end up at exactly midway between the targets due to maximal uncertainty. Contrary to this assumption, in a movement that is time and space constrained, the amount of stochastic evidence is limited and one side would be favored. We tested the model using the parameter values in Fig 4 and simulated trials with equal number on both sides (number ratio = 1). Interestingly, in these trials average reach is dominantly tilted to one side (S6 Fig, A). To fully arrive to target subjects could produce a top-down signal and satisfy. For example, as the index finger gets closer to the computer screen they settle towards the closest target. Satisficing strategies are well known in decision-making literature [66] and can be implemented in the model with a simple extension to Eq (9), weightedq˙=certaintydistance×q˙(13) distance is the normalized depth distance to the screen (0 screen arrival and 1 start button). As the finger approaches the screen the pull exerted by certainty becomes weaker and the movement is mostly driven by the kinematic update q˙ (e.g. at screen arrival: certainty0 = 1). Physical distance is not changing certainty levels, just how relevant it is for updating motor positions. To observe what human participants do and test [file pone.0195188.s006.pdf]

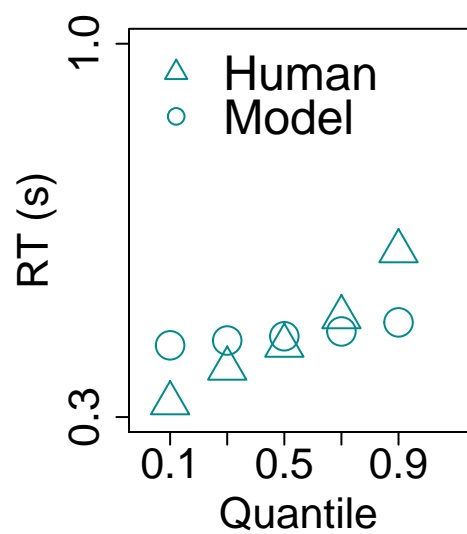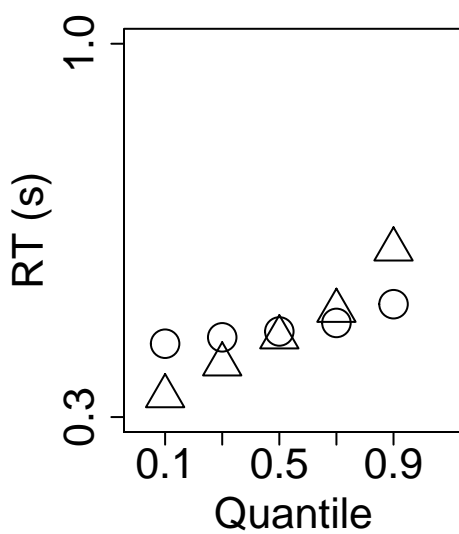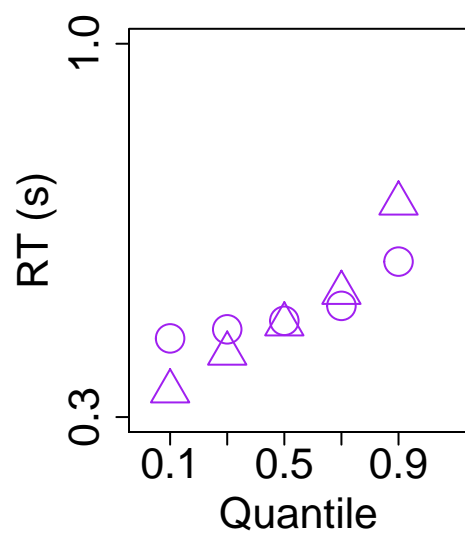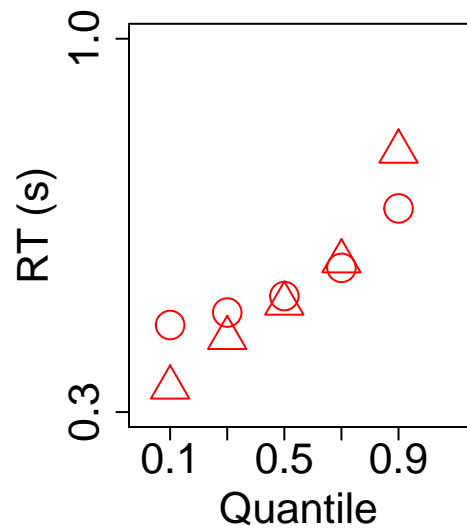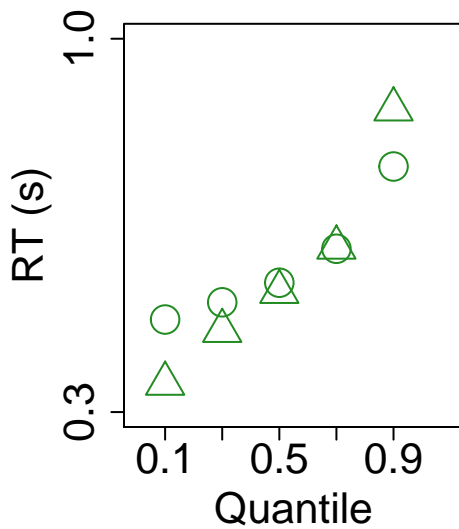

Supplement: S7 Fig — The model reproduces fairly well response times (quantiles 0.25, 0.5, and 0.75) but the distribution of RT for subjects is noisier (see extreme quantiles 0.1 and 0.9). We argue that such additional noise may be in part explained by muscle forces or flight time estimations which were not explicitly included in the model and could have been affected by certainty levels. Ratio 0.1: cyan; 0.25: black; 0.5: purple; 0.75: red; 0.9: green. (PDF) [file pone.0195188.s007.pdf]

Mean RT (s)

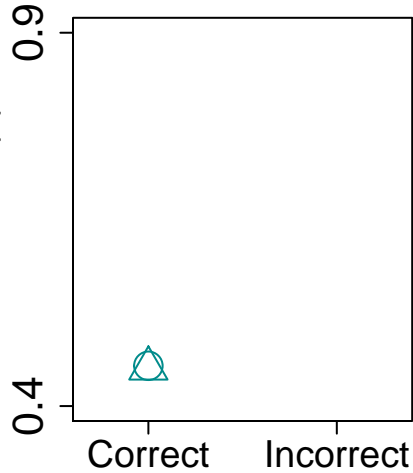

Mean RT (s)

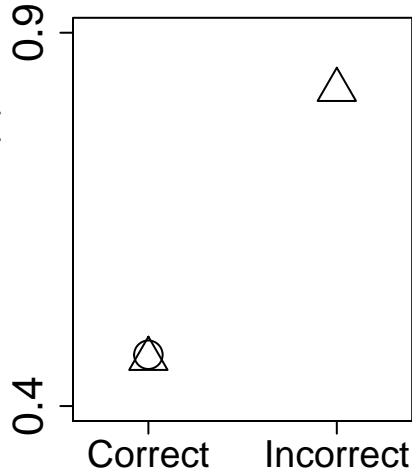

Mean RT (s)

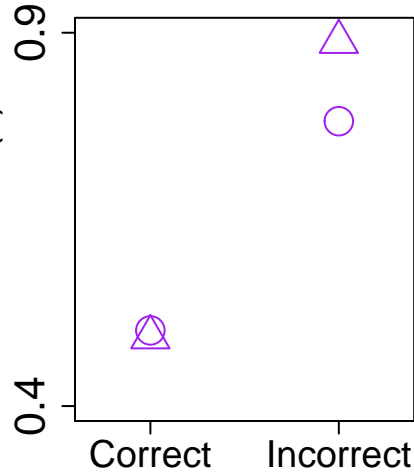

Mean RT (s)

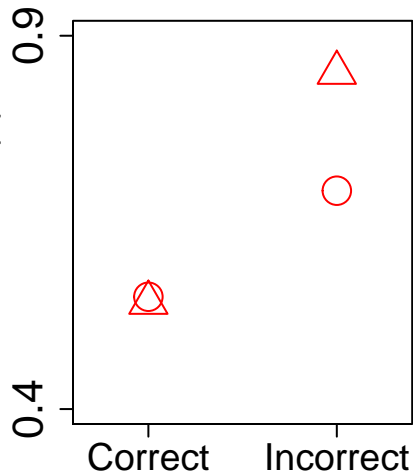

Mean RT (s)

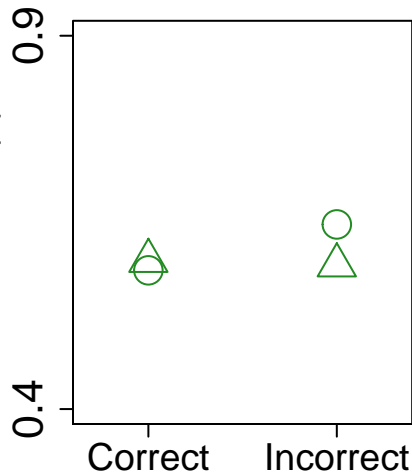

Mean RT (s)

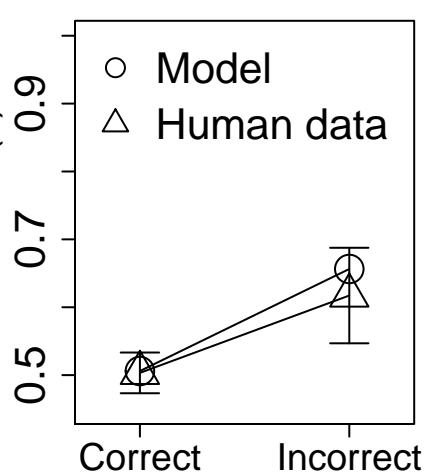

Supplement: S8 Fig — Similarly to human participants, the model was slower in incorrect trials. Ratio 0.1: cyan; 0.25: black; 0.5: purple; 0.75: red; 0.9: green. Lower right panel is overall mean across ratios (F(1,21) = 6.10, p = 0.02, ηg2=0.05). Error bars are s.e.m. (PDF) [file pone.0195188.s008.pdf]

**Human data**

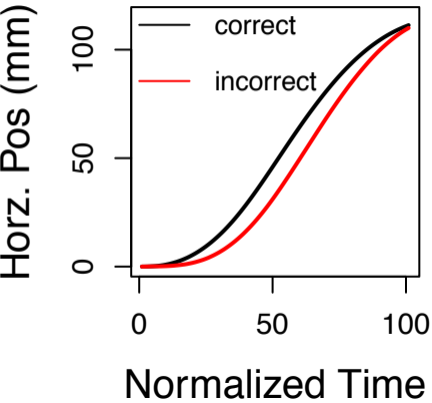

**R: 32; W: 7.72; D: 0.06**

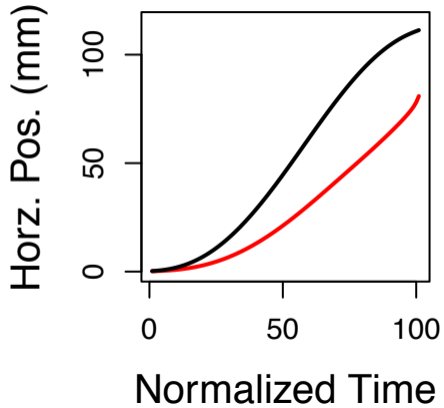

Supplement: S9 Fig — Mean reach in correct and incorrect trials of humans (left panel) and the best fit model (right panel). Incorrect trials are more medial in the model than in humans. This is similar to what happens in trials with the same numerosity on each side (S6 Fig): Evidence quality is weak and hinders arrival to the target. We reasoned that humans may be using a top-down signal to hit the target. See a plausible implementation of such top-down signal in S6 Fig i.e. distance to the screen is used as a cue to stop relying in evidence and instead try to arrive to target (Eq 10). (PDF) [file pone.0195188.s009.pdf]

**Human reach**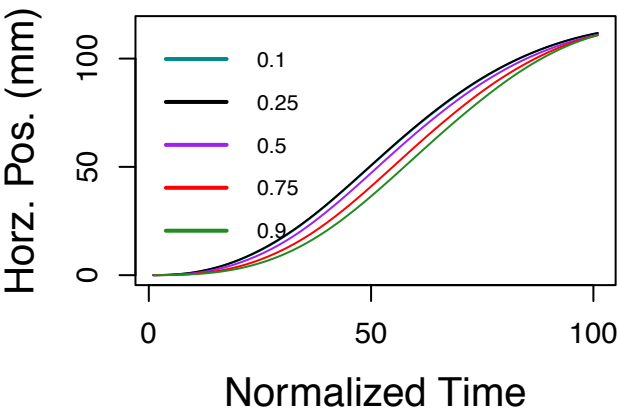**Thr: 0.62; CoM\_Thr: 1.02; Dft: 0.28**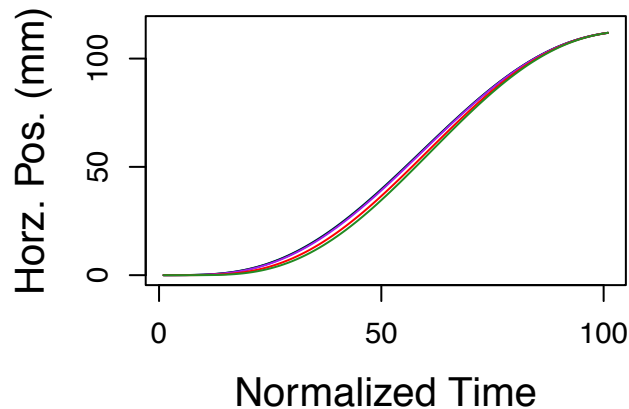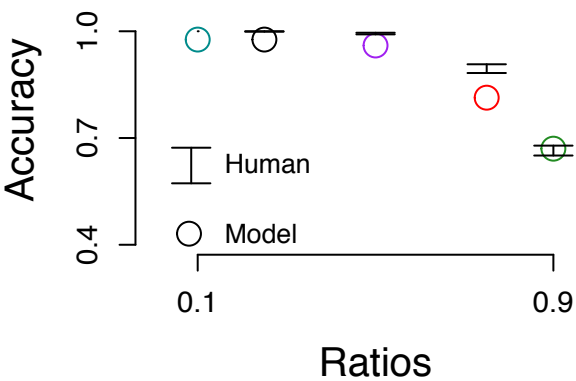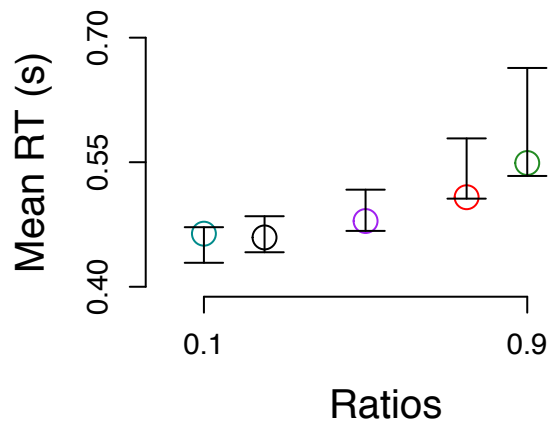

Supplement: S10 Fig — Adding a second post lift-off threshold and a deadline to stop accumulation, as in [15] did not improve motor positioning of the traditional threshold model. On average, they collapsed to a similar trajectory (top right). After the second threshold the model makes a definitive commitment and on average the finger goes directly to target. The fitting procedure could not find a better solution. Best parameters: thr: 0.62, 2nd thr: 1.02, deadline: 51 ms; dft: 0.28; ndt: 0.19 s; sig2: 0.14; k: 3.56. (PDF) [file pone.0195188.s010.pdf]

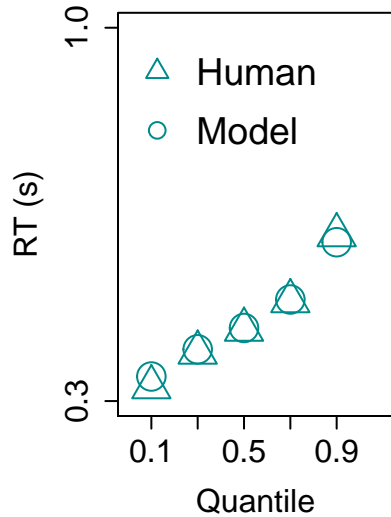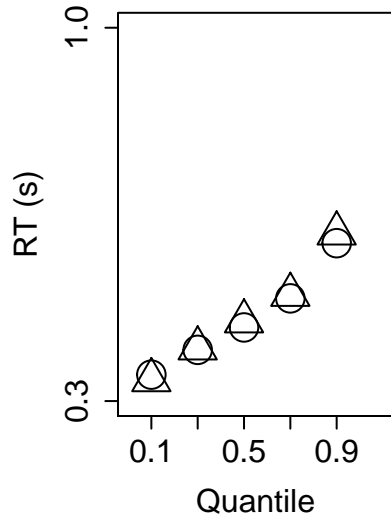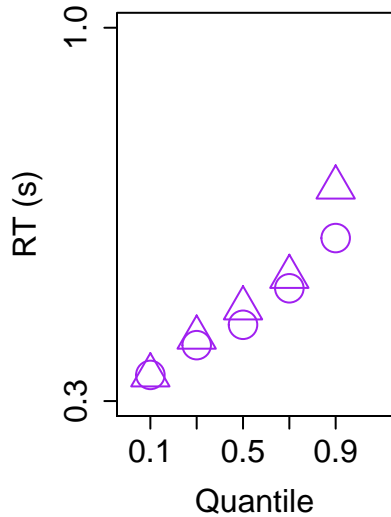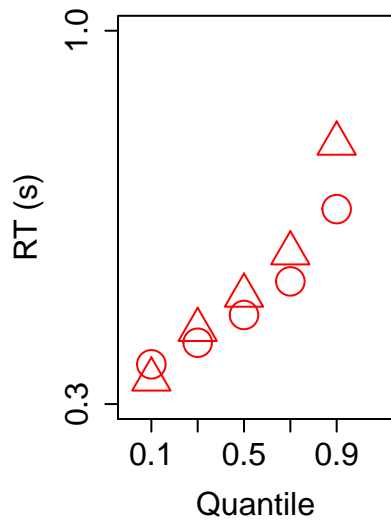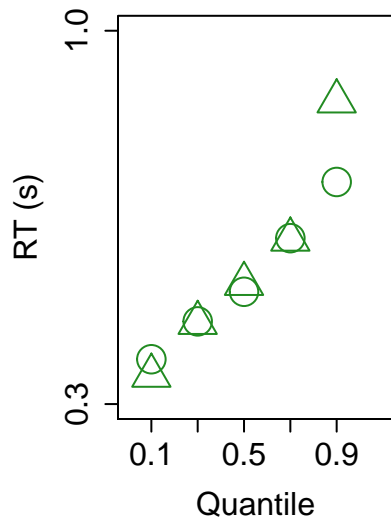

Supplement: S11 Fig — Traditional threshold models are excellent at reproducing response times. (PDF) [file pone.0195188.s011.pdf]
